# Quality of life following total neoadjuvant therapy for rectal cancer

**DOI:** 10.1007/s00432-025-06347-y

**Published:** 2025-10-25

**Authors:** Georg W. Wurschi, Markus Diefenhardt, Justus Kaufmann, Hai Minh Ha, Melanie Schneider, Daphne Schepers von Ohlen, Maren Schöneich, Adrianna Cieslak, Alina Depardon, Jan-Niklas Becker, Alexander Rühle, Felix Ehret, Maximilian Römer, Florian Rißner, Andreas Hinz, Klaus Pietschmann

**Affiliations:** 1 Department of Radiotherapy and Radiation Oncology, Jena University Hospital, Am Klinikum 1, 07747 Jena, Germany; 2 Comprehensive Cancer Center Central Germany (CCCG), Campus Jena, Jena, Germany; 3 Interdisciplinary center for clinical studies (IZKF), Jena University Hospital, Jena, Germany; 4 Department of Radiotherapy and Oncology, University Hospital, Goethe University Frankfurt, Frankfurt, Germany; 5 Frankfurt Cancer Institute (FCI), Frankfurt, Germany; 6 Department of Radiooncology, University Medical Center of the Johannes-Gutenberg-University Mainz, Mainz, Germany; 7 Department of Radiation Oncology, Otto Von Guericke Universität Magdeburg, Magdeburg, Sachsen-Anhalt Germany; 8 Department of Radiotherapy and Radiation Oncology, Faculty of Medicine and University Hospital Carl Gustav Carus, Technische Universität Dresden, Dresden, Germany; 9 Department of Radiotherapy, University Medical Center Schleswig-Holstein/Lübeck, Lübeck, Germany; 10 Department of Radiation Oncology, University Hospital Schleswig-Holstein Campus Kiel, Kiel, Germany; 11 Department of Radiation Oncology, University Medicine Mannheim, Medical Faculty Mannheim, Heidelberg University, Mannheim, Germany; 12 Department of Radiation Oncology, Universitätsklinikum Erlangen, Friedrich-Alexander-Universität Erlangen-Nürnberg, Erlangen, Germany; 13 Department of Radiotherapy, Hannover Medical School, Hannover, Germany; 14 Department of Radiation Oncology, Medical Center – University of Freiburg, Faculty of Medicine, University of Freiburg, German Cancer Consortium (DKTK), Partner Site DKTK-Freiburg, Germany; 15 Department of Radiation Oncology, University Medical Center Leipzig, Leipzig, Germany; 16 Comprehensive Cancer Center Central Germany (CCCG), Campus Leipzig, Leipzig, Germany; 17 Department of Radiation Oncology, Charité– Universitätsmedizin Berlin, Corporate Member of Freie Universität Berlin and Humboldt-Universität Zu Berlin, Berlin, Germany; 18 German Cancer Consortium (DKTK), Partner Site Berlin, A Partnership Between DKFZ and Charité – Universitätsmedizin Berlin, Berlin, Germany; 19 Center for clinical studies, Jena University Hospital, Jena, Germany; 20 Department of Medical Psychology and Medical Sociology, University Medical Center Leipzig, Leipzig, Germany

**Keywords:** Rectal cancer, Total neoadjuvant therapy, Organ preservation, Quality of life, HRQoL, QLQ-CR29, QLQ-C30

## Abstract

**Purpose:**

This study aimed to assess the health-related quality of life (HRQoL) in patients with locally advanced rectal cancer (LARC) undergoing total neoadjuvant therapy (TNT), comparing outcomes with the German general population and colorectal cancer (CRC) patients treated with curative intent.

**Methods:**

In a multicenter, cross-sectional study within the “TNTox” study framework (DRKS 00033000), EORTC QLQ-C30 and QLQ-CR29 questionnaires were distributed to LARC patients who had completed TNT. Mean reference values were compared descriptively, and further exploratory comparisons based on clinical features were performed.

**Results:**

The study included responses from 72 patients. Compared to the German general population, a reduction in mean HRQoL across most domains was observed; the strongest effect was observed for role functioning (− 28.7 points, Cohen’s d = − 0.95), social functioning (− 25.3 points, d = − 0.89), and for diarrhea (+ 9.9 points, d = 0.80). General HRQoL was similar to that of CRC patients following curative treatment. However, some symptom scores, notably fecal incontinence (+ 13.4 points, d = 0.52), impotence (+ 29.0 points, d = 0.73), and dyspareunia (+ 10.4 points, d = 0.40) appeared to be higher. Significant factors associated with HRQoL included the presence of chronic treatment-related toxicity and duration of TNT; no major differences were observed between patients with or without NOM or stoma.

**Conclusion:**

LARC patients undergoing TNT showed comparable HRQoL outcomes to CRC patients treated with curative intent, but with reductions when compared to the general population. The presence of chronic toxicity significantly impacts HRQoL. Survivors may experience HRQoL impairments post-TNT, underscoring the necessity for ongoing management of chronic toxicity tailored to their needs.

**Supplementary Information:**

The online version contains supplementary material available at 10.1007/s00432-025-06347-y.

## Introduction

The prognosis of locally advanced rectal cancer (LARC) has substantially improved by modern resection techniques and emerging multimodal treatment approaches within the last decades (Glynne-Jones et al. 2017; Keller et al. 2020). Within these patients, total neoadjuvant therapy (TNT) is currently widely applied in the presence of certain risk factors, improving both local control and reducing metastatic spread (Bahadoer et al. 2021; Conroy et al. 2021; Fokas et al. 2019; Garcia-Aguilar et al. 2022). This treatment intensification adds preoperative chemotherapy to neoadjuvant radiotherapy (RT) and further increases complete response (CR) rates to 25–65% compared to 10–15% after standard neoadjuvant chemoradiotherapy (CRT) (Kasi et al. 2020; Rödel et al. 2015; Wurschi et al. 2023), enabling non-operative management (NOM) in an increasing number of patients with complete response. Radical resection is known to be associated with reduced health-related quality of life (HRQoL) (Burch et al. 2021). NOM might be associated with better QoL through preserved (anorectal) organ function (Gilbert et al. 2022; Jones et al. 2020). Similarly, a diverting ostomy might deteriorate QoL (Lawday et al. 2021). However, the toxicity profile after TNT varies due to more intense chemotherapy treatment, probably affecting QoL. Chemotherapy within TNT is usually based on 5-fluorouracil and oxaliplatin containing regimens, such as FOLFOX or CAPOX (Ochiai et al. 2024). Oxaliplatin may cause persistent peripheral neurological adverse effects, such as polyneuropathy (PNP) or autonomic dysregulation, relevantly impairing instrumental activities of daily living and QoL (Jordan et al. 2020; Loprinzi et al. 2020).

Given the relatively good prognosis of LARC patients after TNT with 5-year overall survival rates > 74% (Bahadoer et al. 2021; Garcia-Aguilar et al. 2022; Petrelli et al. 2020; Fokas et al. 2022), a relevant proportion of patients might face long-term toxicity that could potentially impact their QoL. It is crucial to identify potential factors that may impair HRQoL in these patients. To date, only limited data on HRQoL are available for this new therapy (Baird et al. 2023). NOM omits radical resection, thus requiring more frequent follow-up visits. It could, on the other hand, also result in increased (psychological) stress due to uncertainty and anxiety regarding tumor control (Liu et al. 2021). For patients following radical resection of colorectal cancers, an improved HRQoL, known as ‘post-traumatic growth’, is described (Kim et al. 2021; Scherer-Trame et al. 2022). Whether this has an inverse effect on HRQoL in the NOM cohort has not been reported so far.

We thus aim to analyze a cross-sectional cohort of LARC patients after TNT within the so-called “TNTox” study (Wurschi et al. 2024) and compare their quality of life with reference data from the German general population and colorectal cancer patients treated with curative intent. In addition, we sought to assess clinically relevant factors potentially associated with QoL.

## Material and methods

We conducted a multicenter analysis within the “Young DEGRO” working group of the German Society for Radiation Oncology (DEGRO) at 23 hospitals in Germany and Austria (Wurschi et al. 2024). During follow-up, the European Organisation for Research and Treatment of Cancer Quality of Life questionnaires QLQ-C30 (EORTC QLQ-C30) (Aaronson et al. 1993) and EORTC QLQ-CR29 (Whistance et al. 2009; Gujral et al. 2007) were distributed prospectively to all eligible patients at 10/23 participating centers, using a cross-sectional approach. Written informed consent was obtained from all participants. The study was approved by the local ethics committee of the Faculty of Medicine, Jena University Hospital (2023-3042-Bef), and by each participating center's ethics committee and adhered to the Declaration of Helsinki. The trial protocol was prospectively registered with the German Clinical Trials Registry (DRKS, No. 00033000) and accredited by the Radiation Oncology Working Group of the German Cancer Society (Arbeitsgemeinschaft Radiologische Onkologie, ARO). All analyses are conducted according to the STROBE criteria (Elm et al. 2007).

Eligible patients were diagnosed with localized rectal cancer (T2-4 N0-2 M0) and underwent neoadjuvant RT, consisting of short-course radiotherapy (SCRT) or long-course chemoradiotherapy (LCRT), followed by consolidation chemotherapy with curative intent between 2015 and 2024. Clinical data were collected retrospectively. Follow-up data were gathered through routine oncological follow-up visits according to institutional standards. Data collection and management were performed using REDCap electronic data capture tools (Harris et al. 2019, 2009) hosted at Jena University Hospital, Jena, Germany.

### Questionnaires

The EORTC QLQ-C30 includes 30 questions that are assigned to five functioning scales (physical, role, cognitive, emotional, and social functioning), three symptom scales (fatigue, pain, and nausea and vomiting), a global health status scale and additional single items assessing commonly reported symptoms (dyspnea, loss of appetite, insomnia, constipation and diarrhea, financial impact of the disease). The QLQ-CR29 module comprises 29 additional specific questions for colorectal cancer patients on 4 multi-item scales and 18 single-items (Whistance et al. 2009), which were subsumed to 4 functioning scales (anxiety, weight loss, body image, sexual interest) and 17 symptom scales (urinary frequency, urinary incontinence, dysuria, abdominal pain, buttock pain, bloating, blood/mucus in stool, dry mouth, hair loss, taste alteration, impotence/dyspareunia, flatulence, fecal incontinence, stool frequency, sore skin, embarrassment). These subscales (range, 0–100) were calculated for both questionnaires (from the corresponding single item values) according to the EORTC manuals (Fayers et al. 2001). Higher scores on functioning scales represent better function, whereas higher symptom scores indicate a high level of symptoms (Aaronson et al. 1993). QLQ-C30 subscales were summarized in the established “Summary Score” (Giesinger et al. 2016). For the QLQ-CR29, no symptom-related summarization of single items is officially validated (Hout et al. 2019). To ease comparison, the items 49–54 were summarized to the “Defecation/Stoma-related problems” scale, which was introduced and internally validated by Stiggelbout et al. (2016).

### Endpoints and definitions

The above-defined QLQ-C30 and QLQ-CR29 subscales served as co-primary endpoints. Given the large number of single-item scales, explorative testing was limited to the most relevant multi-item scales reflecting general QoL, i.e., the “Global Health Scale”, the “Summary Score”, as well as bowel-related symptoms (i.e., “Defecation/Stoma-related problems” and “Blood/mucus in stool”) together with the related “Body image” scale.

Within the prospective study protocol, clinical features, including demographic factors, such as age, sex, presence of chronic treatment-related toxicity, the Karnofsky performance status (KPS), the duration of TNT in months, recurrent disease, as well as NOM and colostomy, were prespecified as factors for further explorative comparison. Chronic toxicity was assessed according to CTCAE v5.0 and was considered symptomatic if graded ≥ 2. For these analyses, all patients undergoing a “watch and wait” approach after CR or local excision were considered as “NOM” patients.

### Statistical analysis

All analyses are exploratory. HRQoL values were primarily reported descriptively with mean (± standard deviation, SD) and median (first and third quartiles). Mean scores of the subscales were compared against reference values for the German general population (Hinz et al. 2014) and EORTC reference values for colorectal cancer (CRC) patients after treatment with curative intent (Whistance et al. 2009). To compare the patients’ scores with those of the general population, we used normative data taken from a large representative general population study for the QLQ-C30 (Hinz et al. 2014). The following strategy was used to mitigate demographic differences regarding sex and age distribution: In the publication of the normative scores, we used the women’s and men’s mean scores and standard deviations of the age decades as reported in the normative table. The age decades were weighted according to the frequencies of the age decades in the cancer sample. For example, the percentage of the four male patients in the age group 40–49 years was 5.6% in this study. These percentages were taken as the weighting factors for calculating the general population mean value of the QLQ-C30 scores.

A difference in mean values of ≥ 10 points was considered clinically relevant (Osoba et al. 1998; Musoro et al. 2020). The effect size for these comparisons was estimated with Cohen’s d (New and Jersey 1988). Cohen’s d > 0.2 was considered as weak, d > 0.5 as moderate, and d > 0.8 as a strong effect, respectively (New and Jersey 1988; Sullivan and Feinn 2012).

Spearman’s correlation was calculated for the evaluation of continuous variables, whereas known group comparisons were performed with Mann–Whitney U tests. Following standard practice (Hout et al. 2019), these comparisons were limited to the Global Health Status (GHS) and the QLQ-C30 summary score (Giesinger et al. 2016) of the QLQ-C30 questionnaire. For the QLQ-CR29, the multi-item scales “Body Image” and “Blood/Mucus in stool” were used, along with “Defecation/Stoma-related problems” (items 49–54). Internal consistency of the latter multi-item scale was assessed using Cronbach’s α and McDonald’s ω prior to the application.

All 95% confidence intervals (95% CI) were obtained via nonparametric bootstrapping with 1000 resamples using the percentile method. Given the exploratory nature of this study, primary analyses were performed using unadjusted two-sided p-values with a significance threshold of *p* < 0.05. To address the issue of multiple testing, test families were defined a priori, and Holm’s sequential Bonferroni procedure was applied within each known-group comparison across the respective 5 HRQoL scales as a sensitivity analysis (adjusted threshold *p* < 0.01). All analyses were conducted with SPSS 29.0 (IBM SPSS Statistics, Armonk, NY / USA) as well as JASP v0.19.3 (JASP Team 2025). Excel v16.96 (Microsoft Inc., Redmond, WA / USA) was used for further visualization.

## Results

### Patient’s characteristics and treatments

For this analysis, questionnaires from 72/295 patients (24.4%) across 10 centers participating (Supplement S1) in the TNTox study were available. Their median age at diagnosis was 65 years (Q1-Q3: (56.5–69.0)), and among them, 54 (75.0%) were men. LCRT was applied in the majority of the patients (66/72, 91.7%). A median of 6.0 (Q1-Q3: 3.0–9.0) cycles of consolidation chemotherapy (standardized to FOLFOX-cycles, i.e., q2w) was administered with a median TNT-duration of 4.4 (Q1-Q3: 2.9–5.4) months. The questionnaires were obtained after a median interval of 17.0 (Q1-Q3: 9.3–34.0) months after completion of TNT. At that time, 26 out of 71 patients (16.9%) presented with a diverting ostomy, and 20 out of 72 patients (27.8%) were managed non-operatively. A detailed description of the characteristics is provided in Tables 1A and 1B. Additional information regarding treatment failures with related treatments and chronic toxicity at follow-up can be found in the Supplements S2 and S3. Overall, symptomatic chronic toxicity (CTCAE grade ≥ 2) was reported for 34 out of 69 (49.3%) patients. Most frequent CTCAE grade ≥ 2 events comprised PNP in 19 out of 69 (27.5%) patients and chronic fatigue in 9 out of 68 (13.2%) patients. Furthermore, CTCAE grade ≥ 2 erectile dysfunction was reported in 14 out of 45 (31.1%) men and dyspareunia in one out of 15 (6.7%) women.

**Table 1:** Characteristics of included patients. Categorical variables (A) and continuous variables (B) with mean and standard deviation (SD) and median, 1st and 3rd quartile, are shown separately. Due to rounding, percentages may not add up to exactly 100%. Note that confidence intervals are based on 1000 bootstrap replicates

Variable	N	n (%)				
(A) *Categorical variables*						
Sex	72	Male: 54 (75.0%)		Female: 18 (25.0%)		
Chemoradiotherapy (LCRT)	72	Yes: 66 (91.7%)		No: 6 (8.3%)		
Non-operative management (NOM)	72	Yes: 20 (27.8%)		No: 52 (72.2%)		
Diverting ostomy at follow-up (“stoma”)	71	Yes: 26 (36.6%)		No: 45 (63.4%)		
Relapse	71	Yes: 12 (16.9%)		No: 59 (83.1%)		
ESMO Risk Classification	72	Early: 1 (1.4%)	Intermediate: 22 (30.6%)	Bad: 15 (20.8%)	Advanced: 34 (47.2%)	
TNM: T Stage	72	T1: 2 (2.8%)	T2: 3 (4.2%)	T3a/b: 36 (50.0%)	T3c/d: 21 (29.2%)	T4: 10 (13.9%)
TNM: N Stage	72	N0: 8 (11.1%)	N1: 26 (36.1%)	N2: 27 (37.5%)	N + (not specified): 11 (15.3%)	
TNM: M Stage	72	M0: 72 (100%)				
Localization from the anal verge	72	0–6 cm 38 (52.8%)	6–12 cm 32 (44.4%)		> 12 cm 2 (2.8%)	
Infiltration of the mesorectal fascia (MRF +)	70	< 1 mm / MRF + 33 (47.1%)	1–2 mm / MRF threatened 9 (12.9%)		> 2 mm / MRF clear 28 (40.0)	
Extramural vascular invasion (EMVI)	51	Yes: 14 (27.5%)		No: 37 (72.5%)		
Involved lateral pelvic nodes	64	Yes: 13 (20.3%)		No: 37 (79.7%)		
Any chronic toxicity at follow-up (CTCAE grade)*	69	≥ 2		34 (49.3%)		
		≥ 3		18 (26.1%)		

	n	Mean	95% Confidence Interval Mean		SD	Median	Q1	Q3
			Lower	Upper				
(B) *Continuous Variables (N = 72)*								
Age (years)	72	63.3	61.0	65.4	9.5	65.0	57.5	69.0
TNT duration (months)	72	4.4	4.1	4.8	1.5	4.4	2.9	5.4
Follow-up (months since end of TNT)	72	22.4	18.3	26.8	18.0	17.0	9.8	34.0
Chemotherapy cycles (standardized to FOLFOX q2w)	72	6.0	5.5	6.7	2.7	6.0	3.0	9.0
Karnofsky Performance Status (KPS): Baseline	72	91.4	89.7	92.9	7.0	90.0	90.0	100.0
Karnofsky Performance Status (KPS): Follow-up	65	86.0	84.0	88.2	8.6	90.0	80.0	90.0

*A detailed description of the distinct toxicity grades is provided in Supplement 3

### Quality of life scores

HRQoL scores are provided for every subscale of the QLQ-C30 and QLQ-CR29 questionnaires in Table 2. A detailed description of the QLQ-CR29 single items for patients with and without a stoma is provided in Supplement S4. The QLQ-CR29 multi-item scale “Defecation/Stoma related problems” was calculated according to Stiggelbout et al. (2016). We found a good internal consistency with Cronbach’s α of 0.847 (95% CI: 0.771–0.901) and McDonald’s ω of 0.845 (95% CI: 0.758–0.899) for this scale in our cohort (Supplement S5). Moderate correlations with most of the established QLQ-C30 and QLQ-CR29 scales related to bowel function/defecation symptoms were observed (Spearman’s ρ 0.45–0.59). Only the correlation with the “Blood and Mucus in stool” scale was not statistically significant (*p* = 0.068). For details, see Supplement S5. Given its good internal consistency and clinically plausible correlations, this new multi-item scale was deemed appropriate for our cohort and was subsequently used for further analyses.

**Table 2:** Summarized QLQ-C30 scores (A) and QLQ-CR29 scores (B) of functioning scores and symptom scores. Descriptive parameters, i.e., the number of questionnaires (n), mean together with standard deviation (SD), and 95% confidence interval (CI), are provided (B). 95% CIs are based on 1000 bootstrap samples. Higher scores in function scales indicate better functioning, whereas higher scores in symptom scales indicate more symptoms

		Mean	95% Confidence Interval Mean		SD	Median	Q1	Q3
	n		Lower	Upper				
(A) *QLQ-C30 Scores*								
Functioning scales								
Physical Functioning	72	76.7	70.9	81.6	23.6	80.0	60.0	100.0
Role Functioning	70	61.7	54.3	68.6	31.6	66.7	33.3	95.8
Emotional Functioning	70	75.4	68.3	81.3	26.7	83.3	58.3	100.0
Cognitive Functioning	69	82.1	76.3	87.2	23.3	100.0	66.7	100.0
Social Functioning	68	68.1	60.8	75.0	31.2	66.7	50.0	100.0
Global Health	69	64.1	58.6	69.2	23.1	66.7	50.0	83.3
Summary Score	64	77.8	73.0	82.3	19.0	81.8	64.5	92.5
Symptom scales								
Fatigue	71	29.0	23.2	35.1	26.4	22.2	5.6	44.4
Nausea/Vomiting	70	5.0	2.6	8.3	12.2	0.0	0.0	0.0
Pain	70	19.8	14.3	26.0	25.9	8.3	0.0	33.3
Dyspnea	71	16.9	12.2	22.5	23.8	0.0	0.0	33.3
Insomnia	71	28.6	21.1	36.6	33.5	33.3	0.0	66.7
Appetite Loss	71	13.1	6.6	19.3	27.9	0.0	0.0	0.0
Constipation	71	13.1	7.5	19.2	24.9	0.0	0.0	33.3
Diarrhea	69	21.3	14.0	29.0	30.8	0.0	0.0	33.3
Financial Difficulties	68	17.6	11.3	24.5	29.1	0.0	0.0	33.3
(B) *QLQ-CR29 Scores*								
Functioning scales								
Body Image	69	77.0	71.3	82.9	25.6	88.9	66.7	100.0
Weight Loss	69	78.3	72.0	84.1	25.5	100.0	66.7	100.0
Anxiety	69	60.4	52.7	68.6	33.0	66.7	33.3	100.0
Sexual Interest (Men)	48	42.4	34.0	50.7	32.1	33.3	25.0	66.7
Sexual Interest (Women)	15	22.2	11.1	35.6	24.1	33.3	0.0	33.3
Defecation/Stoma-related problems	64	29.7	24.6	35.5	23.1	27.8	11.1	44.4
Symptom scales								
Urinary Frequency	69	37.9	31.9	43.7	25.9	33.3	16.7	50.0
Urinary Incontinence	67	11.9	7.5	15.9	19.0	0.0	0.0	33.3
Dysuria	71	7.5	3.8	12.2	18.9	0.0	0.0	0.0
Abdominal Pain	71	11.7	7.0	16.4	20.4	0.0	0.0	33.3
Buttock Pain	71	23.0	17.4	28.6	26.8	0.0	0.0	33.3
Bloating	70	21.4	15.2	28.1	26.6	0.0	0.0	33.3
Blood/Mucus in Stool	65	7.9	5.1	11.0	12.9	0.0	0.0	16.7
Dry Mouth	71	16.9	11.3	22.5	24.5	0.0	0.0	33.3
Hair Loss	71	16.0	10.3	22.1	25.7	0.0	0.0	33.3
Taste Alteration	71	15.5	8.9	23.0	29.2	0.0	0.0	33.3
Impotence (Men)	46	63.0	51.4	73.9	38.6	66.7	33.3	100.0
Dyspareunia (Women)	12	19.4	5.6	36.1	30.0	0.0	0.0	33.3
Stoma Care Problems	25	12.0	4.0	21.3	23.3	0.0	0.0	33.3
Flatulence	65	33.8	27.2	41.0	29.8	33.3	0.0	33.3
Fecal Incontinence	63	25.4	18.5	32.8	28.5	33.3	0.0	33.3
Sore Skin	63	26.5	19.0	34.4	31.2	33.3	0.0	33.3
Stool Frequency	62	31.5	25.0	38.7	27.2	33.3	4.2	50.0
Embarrassment	63	31.7	22.8	41.8	38.1	0.0	0.0	66.7

### Comparison with reference values

**Fig. 1:**
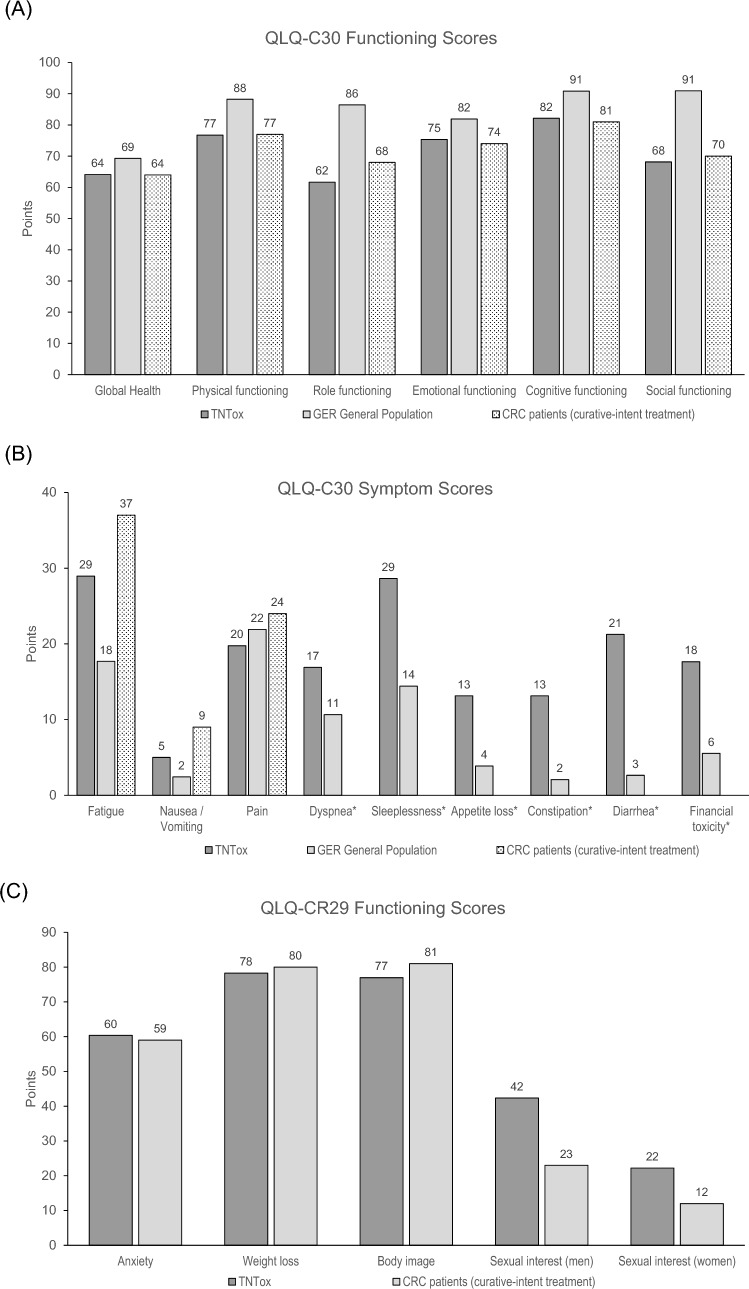

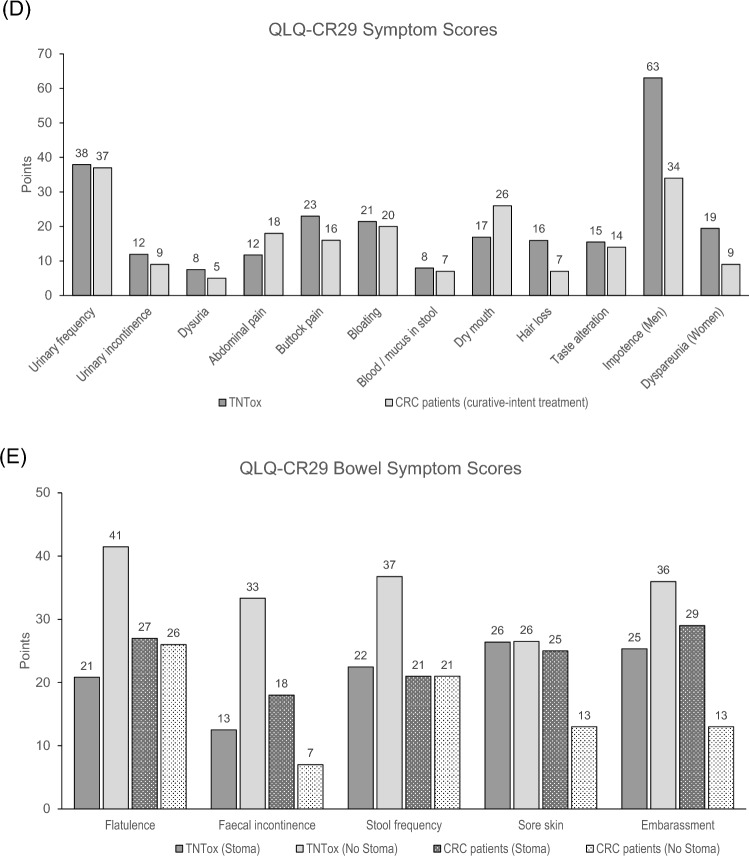
Mean values of quality of life (HRQoL) of the TNTox cohort, EORTC reference values for colorectal cancer (CRC) patients after curative-intent treatment (Whistance et al. 2009), and reference values for the German general population for QLQ-C30 (**A** + **B**) and QLQ-CR29 (**C**–**E**) subscales. QLQ-CR29 Bowel Symptoms are stratified per presence of a stoma for the TNTox cohort and for the CRC cohort. Higher values in the functioning scales (**A** + **C**) and lower values in the symptom scales (B, D, E) indicate better HRQoL in the respective subscales. *No values were available for the CRC cohort in these subdomains

Displays the respective mean values together with published reference data from the German general population, standardized for age and sex distribution, and the EORTC validation cohort of CRC patients after curative-intent treatment. The weighting of the German reference values by age and sex is detailed in Supplement S6. Supplement S7 provides a comprehensive overview of the mean value comparisons shown in Fig. 1. Overall, the mean HRQoL values were lower compared with the German general population’s functioning scores (d = − 0.27 to d = − 0.95). Similarly, mean symptom scores were higher in our cohort (d = 0.08 to d = 0.80). We found a small effect regarding the “Global Health Scale” (− 5.2 points, d = − 0.27). The strongest effects (d ≥ 0.8) were observed for role functioning (− 28.7 points, d = − 0.95), social functioning (− 25.3 points, d = − 0.89), and diarrhea (+ 18.7 points, d = 0.80). Of note, mean scores for financial difficulties were higher in the TNT cohort than in the German general population (+ 12.8 points, d = 0.51). Only a marginal difference (d ≤ 0.2) was found for “Pain” (− 2.1 points, d = − 0.08). The EORTC CRC-patient population had comparable QLQ-C30 function scores, with differences in mean values being < 10 points (d < 0.2). Regarding the three available symptom scores, we found lower mean values in our cohort for Fatigue and Nausea/Vomiting (− 12.0 points, d = − 0.30 and − 4.0 points, d = − 0.26, respectively). Comparing the QLQ-CR29 values, we found comparable function scores, but mean sexual interest was higher in our cohort (men: + 19.4 points, d = 0.62; women: + 10.2 points, d = 0.43). Most of the mean symptom scores were slightly higher in our cohort (< 10 points), except for “Fecal Incontinence” (+ 13.4 points, d = 0.52), “Embarrassment” (+ 12.7 points, d = 0.37),”Impotence” in men (+ 29.0 points, d = 0.73) and “Dyspareunia” in women (+ 10.4 points, d = 0.40). In contrast, “Abdominal Pain”, “Dry Mouth”, and “Stoma Care Problems” mean values were slightly lower (− 4.0 to − 9.1 points; d = − 0.18 to − 0.34).

### Subgroup and correlation analyses

**Fig. 2:**
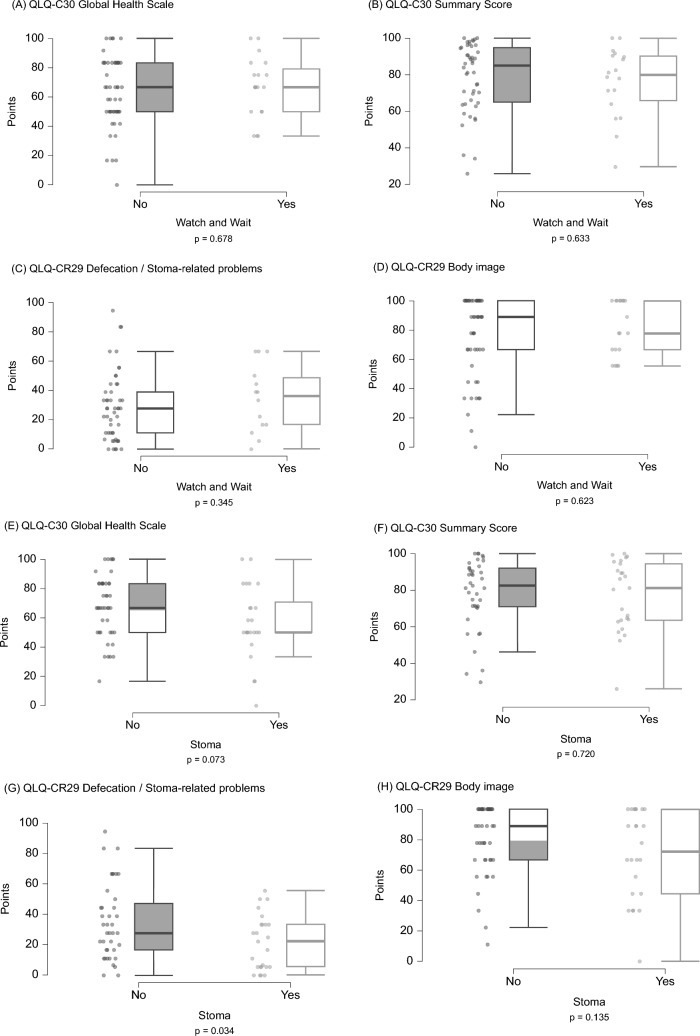
Boxplots of relevant multi-item scales, including the QLQ-C30 “Global Health Status” and “Summary Score”, as well as the QLQ-CR29 scales “Defecation/Stoma-related Problems” and “Body Image”, for known-group comparisons (Watch and Wait, A–D; colostomy, E–H). “Watch and Wait” refers to patients who did not undergo radical surgery at the time of follow-up

We conducted a comparison of the above-defined scales between patients with and without colostomy as well as patients with or without NOM, which is shown in Fig. 2. For both groups, no significant differences were observed, except for “Defecation/Stoma-related Problems” (QLQ-CR29), where stoma patients showed lower ranks (*p* = 0.034, not significant after Bonferroni correction; Fig. 1 and Supplement S4). Furthermore, ranks were not significantly different between men and women. Of note, significantly worse global HRQoL (according to the “Global Health Scale”, *p* = 0.002, and the “Summary Score”, *p* < 0.001) as well as higher “Defecation/Stoma-related Problems” (*p* = 0.003) were observed in patients presenting with symptomatic chronic toxicity, i.e., CTCAE grade ≥ 2. HRQoL ranks were not significantly different in patients who were diagnosed with recurrent disease at follow-up. Detailed test statistics for these known group comparisons are provided in Supplement S8. Furthermore, we found a significant correlation of patients’ age with “Body Image” (Spearman’s ρ = 0.253, *p* = 0.036). The duration of TNT inversely correlated with the “Global Health Scale” (Spearman’s ρ = − 0.251, *p* = 0.037) as well as the QLQ-C30 “Summary Score” (Spearman’s ρ = − 0.276, *p* = 0.027), all not significant after Bonferroni correction. For these scales, a positive correlation with KPS at follow-up was observed (Spearman’s ρ = 0.495, *p* < 0.001 and ρ = 0.436, *p* < 0.001, respectively). Additional correlation analyses are reported in Supplement S9.

## Discussion

We analyzed the health-related HRQoL following TNT in a prospective multicenter cohort in Germany. Our data showed a relevant reduction of HRQoL in most domains in comparison with an age- and sex-corrected cohort of a large prospective reference group from the German general population (N = 2448). However, the HRQoL appeared to be comparable with the initial EORTC validation cohort of CRC patients. These results are of particular interest, as certain HRQoL domains were impaired after rectal cancer treatment in several studies (Giandomenico et al. 2015). TNT is linked to a substantial improvement in clinical outcomes, such as overall survival (Conroy et al. 2021) or increased CR rates (Donnelly et al. 2023; Turri et al. 2024). Some earlier trials have demonstrated an increase of toxicity relative to standard CRT or SCRT, which predominantly involves hematological side effects and bowel-related symptoms, specifically diarrhea (Turri et al. 2024; Seow et al. 2025). Moreover, there have been reports of elevated incidences of fatigue or neurotoxicity, i.e., PNP. However, overall toxicity and treatment-related mortality were not significantly different between standard CRT and different TNT regimes in a network meta-analysis (Seow et al. 2025). Consistent with these reports, we did not observe differences in general HRQoL compared to the EORTC reference cohort, which included CRC patients after different treatments with curative intent. Given the substantial number of long-term cancer survivors after curative-intent treatment of LARC, these findings may be of clinical relevance for treatment planning and shared decision making, as they suggest that the oncological benefits of TNT do not necessarily come at the cost of HRQoL.

Compared to the general population, several studies reported a significant reduction in specific HRQoL domains, although general QoL appeared not to be reduced in a review (Giandomenico et al. 2015). In contrast, we found both reduced global and functional QoL compared to the general population. This observation may not only relate to our primary data but also to the selection of the reference group. We referred to the reference values provided by Hinz et al. (2014), who collected their questionnaires from participants contacted randomly across Germany in face-to-face interviews. Many other studies included in that review obtained their normative data from respondents of internet-based questionnaires, making these samples susceptible to sampling bias (Basch et al. 2018). For example, Nolte et al. (2019) reported nearly 10 points lower HRQoL for most QLQ-C30 domains in a comparable reference cohort of the German general population, which was obtained from internet-based interviews. Referring to our values and those presented by Nolte et al., we would also assume less impact on HRQoL. Secondly, age- and sex-dependent differences in HRQoL have been reported (Hinz et al. 2014, 2019; Nolte et al. 2019, 2020), likely influencing the comparison in the case of demographic differences. We mitigated this latter effect by applying age- and sex-weighted reference values. Thus, the selection of appropriate reference cohorts is crucial and might significantly influence the conclusions of such analyses.

There seemed to be no relevant difference in HRQoL across most domains in our patients stratified by NOM and stoma, except for possibly higher bowel-related symptom burden in patients without a stoma. These results are in contrast with the literature; a better bowel function in NOM-patients has been reported (Bach et al. 2021; Quezada-Diaz et al. 2020) as well as a negative impact of a colostomy on HRQoL (Downing et al. 2019; Vonk-Klaassen et al. 2016). Bowel-related symptom scores appeared higher in our cohort compared to the EORTC reference values, which might be a result of the short time interval after TNT in many patients. Whether this observation is further attributed to differences in treatment intensity or to the fact that NOM patients were more likely to suffer from chronic rectal inflammation cannot be concluded. However, caution is advised in interpreting our results due to the small sample sizes in these subgroups. Restorative surgery, i.e., the closure of a stoma after rectal cancer resection, was not found to improve HRQoL in general in a systematic review (Lawday et al. 2022). In contrast, another review reported reduced HRQoL in patients with stoma-related problems (Vonk-Klaassen et al. 2016). We also found lower ranks of most of the multi-item scores for patients with symptomatic chronic toxicity and, inversely, better HRQoL with increased KPS at follow-up. Although guidelines for the reporting of patient-reported outcomes (PROs), such as PRO-CONSORT or ISOQOL, have been published, a review found substantial inconsistency between clinician and patient reporting of toxicity (as measured with HRQoL) for rectal cancer (Gilbert et al. 2015).

In our cohort, a high grade of erectile dysfunction was reported along with high HRQoL symptom scores in men. Despite the small sample size, these impairments seemed to be more common in men than in women. Impairment of sexual function after rectal cancer treatment is frequently described in the literature for both men and women (Ho et al. 2011; Lim et al. 2014). However, its severity seemed to exceed the values provided by the EORTC CRC cohort. A high cumulative oxaliplatin dose was administered to the patients of our cohort, which led to symptomatic PNP in more than one out of four patients. Oxaliplatin may also cause impairment of vegetative functions, especially erectile function (Loprinzi et al. 2020; Cersosimo 2005). Whether this explains the increased symptom scores and why a relatively lower incidence of toxicity was reported for women cannot be concluded from this analysis. HRQoL data from the PROSPECT trial, in which neoadjuvant CRT was compared with neoadjuvant chemotherapy (FOLFOX), indicate that patients treated with chemotherapy alone experienced less diarrhea and better bowel function than those receiving CRT, but a higher burden of other symptoms (e.g., fatigue, anxiety, nausea, and neuropathy) (Basch et al. 2023). At 12 months, FOLFOX patients reported lower rates of fatigue, neuropathy, and sexual dysfunction. These findings suggest that the more intensive systemic treatment may be associated with a (transient) impairment of HRQoL, while CRT could have a negative impact on long-term HRQoL. As different QoL questionnaires were used, a direct comparison with our cohort was not possible.

The reported financial difficulty scores were higher than in the general population. This observation is in line with a German sample of patients with different cancer types after radiotherapy. In that cohort, 29% of rectal cancer patients reported financial difficulties related to their treatment (Fabian et al. 2025; Wurschi et al. 2024).

## Strengths and limitations

We prospectively collected standardized questionnaires from a multicenter cohort throughout Germany. All patients underwent a comparable treatment sequence, consisting of neoadjuvant RT or CRT, followed by consolidation chemotherapy. The use of the QLQ-C30 and QLQ-CR29 enables comparison with various cohorts; employing age- and sex-weighted reference values from the German general population helped to mitigate the influence of demographic differences. This standardization was not applied to reference values from the EORTC cohort of CRC patients, due to the absence of age and sex subgroups. With a mean age of 65 years and 58% male patients, the demographic influence appeared relatively minor.

However, several limitations must be acknowledged. First, this is a relatively small sample from a non-randomized cohort of patients. HRQoL questionnaires were collected in only 10 of the 23 participating centers, primarily due to local data protection regulations or institutional policies. Consequently, the HRQoL sample does not represent the entire cohort, and the generalizability of the HRQoL findings may be limited. With a median follow-up interval < 18 months, recovery from chronic toxicity, such as fatigue, persistent bowel irritation or neuropathy, may not yet be complete in all patients. This may affect the interpretation of our results in comparison with cohorts with longer follow-up periods (Rausa et al. 2017). Nonetheless, this factor was assessed in exploratory correlation analyses and did not show a significant impact on the multi-item scales in our cohort. As we included patients over a period of nearly one decade, advances in supportive care and their gradual implementation into routine clinical practice must be taken into account. In Germany, the national S3 guideline on supportive care has been available since 2016, and its recommendations may have influenced the management of some patients included in this study. In this context, CRC patients of the EORTC reference group (published in 2009) may not have received current treatment techniques (such as intensity-modulated radiotherapy, IMRT) or the same supportive care measures, which could potentially influence the interpretation of our results.

Due to the small sample size, no complex regression analyses were conducted. Moreover, the cross-sectional approach does not allow for longitudinal comparison or adjustment to baseline values, which would be essential to differentiate between comorbidity-related impairments of HRQoL and treatment-related impairments. Additionally, no information on income or educational status was available for our cohort.

## Conclusion

In our cohort of rectal cancer patients, HRQoL was lower following TNT compared with the German general population, but comparable with most HRQoL subscales of CRC patients. HRQoL was similar in most subgroup analyses regarding NOM and the presence of a stoma. However, patients with symptomatic toxicity at follow-up reported lower HRQoL within the QLQ-C30 questionnaire. These findings highlight the importance of professional healthcare management to maintain quality of life among cancer survivors. Future trials may include QoL endpoints to improve rectal cancer treatment from both the clinician’s and the patients’ perspective.

## Supplementary Information

Below is the link to the electronic supplementary material.

Supplementary file1 (PDF 1292 kb)

## Data Availability

The datasets generated during and/or analyzed during the current study are available from the corresponding author on reasonable request.

## Abbreviations

95% CI: 95% Confidence interval
APC: Argon plasma coagulation
ASCRS: American Society of Colon and Rectal Surgeons
CR: Complete response
CRT: Chemoradiotherapy
CRC: Colorectal cancer
CTCAE: Common terminology criteria of adverse events
DEGRO: Deutsche Gesellschaft für Radioonkologie (German Society for Radiation Oncology)
EMVI: Extramural vascular invasion
EORTC: European Organisation for Research and Treatment of Cancer
ESMO: European Society for Medical Oncology
HRQoL: Health-related quality of life
IMRT: Intensity-modulated radiotherapy
KPS: Karnofsky performance status
LCRT: Long-course chemoradiotherapy
MRF: Mesorectal fascia
NOM: Non-operative management
PRO: Patient-reported outcome
QoL: Quality of life
SCRT: Short-course radiotherapy
TNT: Total neoadjuvant therapy

## References

[CR1] Aaronson NK, Ahmedzai S, Bergman B, Bullinger M, Cull A, Duez NJ et al (1993) The European Organization for Research and Treatment of Cancer QLQ-C30: a quality-of-life instrument for use in international clinical trials in oncology. J Natl Cancer Inst 85(5):365–376. 10.1093/jnci/85.5.365 8433390 10.1093/jnci/85.5.365

[CR2] Bach SP, Gilbert A, Brock K, Korsgen S, Geh I, Hill J et al (2021) Radical surgery versus organ preservation via short-course radiotherapy followed by transanal endoscopic microsurgery for early-stage rectal cancer (TREC): a randomised, open-label feasibility study. Lancet Gastroenterol Hepatol 6(2):92–105. 10.1016/S2468-1253(20)30333-2 33308452 10.1016/S2468-1253(20)30333-2 PMC7802515

[CR3] Bahadoer RR, Dijkstra EA, van Etten B, Marijnen CAM, Putter H, Kranenbarg EMK et al (2021) Short-course radiotherapy followed by chemotherapy before total mesorectal excision (TME) versus preoperative chemoradiotherapy, TME, and optional adjuvant chemotherapy in locally advanced rectal cancer (RAPIDO): a randomised, open-label, phase 3 trial. Lancet Oncol 22(1):29–42. 10.1016/S1470-2045(20)30555-6 33301740 10.1016/S1470-2045(20)30555-6

[CR4] Baird P, Steinke JD, Minnaar HS, Stewart AJ (2023) Assessment of quality of life in rectal cancer with organ-preservation treatment: are we there yet? Clin Oncol (r Coll Radiol) 35(2):e110–e120. 10.1016/j.clon.2022.11.002 36443138 10.1016/j.clon.2022.11.002

[CR5] Basch E, Dueck AC, Rogak LJ, Mitchell SA, Minasian LM, Denicoff AM et al (2018) Feasibility of implementing the patient-reported outcomes version of the common terminology criteria for adverse events in a multicenter trial: NCCTG N1048. J Clin Oncol 36(31):3120–3125. 10.1200/jco.2018.78.8620 10.1200/JCO.2018.78.8620 PMC6209091 30204536

[CR6] Basch E, Dueck AC, Mitchell SA, Mamon H, Weiser M, Saltz L et al (2023) Patient-reported outcomes during and after treatment for locally advanced rectal cancer in the PROSPECT trial (Alliance N1048). J Clin Oncol 41(21):3724–3734. 10.1200/jco.23.00903 37270691 10.1200/JCO.23.00903 PMC10351948

[CR7] Burch J, Taylor C, Wilson A, Norton C (2021) Symptoms affecting quality of life after sphincter-saving rectal cancer surgery: a systematic review. Eur J Oncol Nurs 52:101934. 10.1016/j.ejon.2021.101934 33845303 10.1016/j.ejon.2021.101934

[CR8] Cersosimo RJ (2005) Oxaliplatin-associated neuropathy: a review. Ann Pharmacother 39(1):128–135. 10.1345/aph.1E319 15590869 10.1345/aph.1E319

[CR9] Conroy T, Bosset J-F, Etienne P-L, Rio E, François É, Mesgouez-Nebout N et al (2021) Neoadjuvant chemotherapy with FOLFIRINOX and preoperative chemoradiotherapy for patients with locally advanced rectal cancer (UNICANCER-PRODIGE 23): a multicentre, randomised, open-label, phase 3 trial. Lancet Oncol 22(5):702–715. 10.1016/S1470-2045(21)00079-6 33862000 10.1016/S1470-2045(21)00079-6

[CR10] Donnelly M, Ryan OK, Ryan ÉJ, Creavin B, O’Reilly M, McDermott R et al (2023) Total neoadjuvant therapy versus standard neoadjuvant treatment strategies for the management of locally advanced rectal cancer: network meta-analysis of randomized clinical trials. BJS 110(10):1316–1330. 10.1093/bjs/znad177 37330950 10.1093/bjs/znad177

[CR11] Downing A, Glaser AW, Finan PJ, Wright P, Thomas JD, Gilbert A et al (2019) Functional outcomes and health-related quality of life after curative treatment for rectal cancer: a population-level study in England. Int J Radiat Oncol Biol Phys 103(5):1132–1142. 10.1016/j.ijrobp.2018.12.005 30553942 10.1016/j.ijrobp.2018.12.005

[CR12] Fabian A, Rühle A, Liegl G, Domschikowski J, Trommer M, Ferdinandus S et al (2025) Quality of life in cancer patients at the end of radiotherapy compared to a general population sample in Germany. Int J Cancer. 10.1002/ijc.70152 40938250 10.1002/ijc.70152 PMC12712360

[CR13] Fayers P, Aaronson NK, Bjordal K, Grønvold M, Curran D, Bottomley A (2001) EORTC QLQ-C30 scoring manual. European Organisation for research and treatment of cancer

[CR14] Fokas E, Allgäuer M, Polat B, Klautke G, Grabenbauer GG, Fietkau R et al (2019) Randomized phase II trial of chemoradiotherapy plus induction or consolidation chemotherapy as total neoadjuvant therapy for locally advanced rectal cancer: CAO/ARO/AIO-12. J Clin Oncol 37(34):3212–3222. 10.1200/jco.19.00308 31150315 10.1200/JCO.19.00308

[CR15] Fokas E, Schlenska-Lange A, Polat B, Klautke G, Grabenbauer GG, Fietkau R et al (2022) Chemoradiotherapy plus induction or consolidation chemotherapy as total neoadjuvant therapy for patients with locally advanced rectal cancer: long-term results of the CAO/ARO/AIO-12 randomized clinical trial. JAMA Oncol 8(1):e215445-e. 10.1001/jamaoncol.2021.5445 34792531 10.1001/jamaoncol.2021.5445 PMC8603234

[CR16] Garcia-Aguilar J, Patil S, Gollub MJ, Kim JK, Yuval JB, Thompson HM et al (2022) Organ preservation in patients with rectal adenocarcinoma treated with total neoadjuvant therapy. J Clin Oncol 40(23):2546–2556. 10.1200/jco.22.00032 35483010 10.1200/JCO.22.00032 PMC9362876

[CR17] Giandomenico F, Gavaruzzi T, Lotto L, Del Bianco P, Barina A, Perin A et al (2015) Quality of life after surgery for rectal cancer: a systematic review of comparisons with the general population. Expert Rev Gastroenterol Hepatol 9(9):1227–1242 26197061 10.1586/17474124.2015.1070667

[CR18] Giesinger JM, Kieffer JM, Fayers PM, Groenvold M, Petersen MA, Scott NW et al (2016) Replication and validation of higher order models demonstrated that a summary score for the EORTC QLQ-C30 is robust. J Clin Epidemiol 69:79–88. 10.1016/j.jclinepi.2015.08.007 26327487 10.1016/j.jclinepi.2015.08.007

[CR19] Gilbert A, Ziegler L, Martland M, Davidson S, Efficace F, Sebag-Montefiore D et al (2015) Systematic review of radiation therapy toxicity reporting in randomized controlled trials of rectal cancer: a comparison of patient-reported outcomes and clinician toxicity reporting. Int J Radiat Oncol Biol Phys 92(3):555–567. 10.1016/j.ijrobp.2015.02.021 26068490 10.1016/j.ijrobp.2015.02.021

[CR20] Gilbert A, Homer V, Brock K, Korsgen S, Geh I, Hill J et al (2022) Quality-of-life outcomes in older patients with early-stage rectal cancer receiving organ-preserving treatment with hypofractionated short-course radiotherapy followed by transanal endoscopic microsurgery (TREC): non-randomised registry of patients unsuitable for total mesorectal excision. Lancet Healthy Longevity 3(12):e825–e838. 10.1016/S2666-7568(22)00239-2 36403589 10.1016/S2666-7568(22)00239-2 PMC9722406

[CR21] Glynne-Jones R, Wyrwicz L, Tiret E, Brown G, Rodel C, Cervantes A et al (2017) Rectal cancer: ESMO clinical practice guidelines for diagnosis, treatment and follow-up. Ann Oncol 28(4):iv22–iv40. 10.1093/annonc/mdx224 28881920 10.1093/annonc/mdx224

[CR22] Gujral S, Conroy T, Fleissner C, Sezer O, King PM, Avery KNL et al (2007) Assessing quality of life in patients with colorectal cancer: an update of the EORTC quality of life questionnaire. Eur J Cancer 43(10):1564–1573. 10.1016/j.ejca.2007.04.005 17521904 10.1016/j.ejca.2007.04.005

[CR23] Harris PA, Taylor R, Thielke R, Payne J, Gonzalez N, Conde JG (2009) Research electronic data capture (REDCap)–a metadata-driven methodology and workflow process for providing translational research informatics support. J Biomed Inform 42(2):377–381. 10.1016/j.jbi.2008.08.010 18929686 10.1016/j.jbi.2008.08.010 PMC2700030

[CR24] Harris PA, Taylor R, Minor BL, Elliott V, Fernandez M, O’Neal L et al (2019) The REDCap consortium: building an international community of software platform partners. J Biomed Inform 95:103208. 10.1016/j.jbi.2019.103208 31078660 10.1016/j.jbi.2019.103208 PMC7254481

[CR25] Hinz A, Singer S, Brähler E (2014) European reference values for the quality of life questionnaire EORTC QLQ-C30: results of a German investigation and a summarizing analysis of six European general population normative studies. Acta Oncol 53(7):958–965. 10.3109/0284186X.2013.879998 24456505 10.3109/0284186X.2013.879998

[CR26] Hinz A, Mehnert A, Dégi C, Reissmann DR, Schotte D, Schulte T (2017) The relationship between global and specific components of quality of life, assessed with the EORTC QLQ-C30 in a sample of 2019 cancer patients. Eur J Cancer Care (Engl). 10.1111/ecc.12416 26568527 10.1111/ecc.12416

[CR27] Ho VP, Lee Y, Stein SL, Temple LKF (2011) Sexual function after treatment for rectal cancer: a review. Dis Colon Rectum 54(1):113–125. 10.1007/DCR.0b013e3181fb7b82 21160322 10.1007/DCR.0b013e3181fb7b82

[CR28] JASP Team. JASP (Version 0.19.3). 0.19.3 ed. Amsterdam, The Netherlands

[CR29] Jones HJS, Al-Najami I, Cunningham C (2020) Quality of life after rectal-preserving treatment of rectal cancer. Eur J Surg Oncol 46(11):2050–2056. 10.1016/j.ejso.2020.07.018 32773139 10.1016/j.ejso.2020.07.018

[CR30] Jordan B, Margulies A, Cardoso F, Cavaletti G, Haugnes HS, Jahn P et al (2020) Systemic anticancer therapy-induced peripheral and central neurotoxicity: ESMO-EONS-EANO Clinical Practice Guidelines for diagnosis, prevention, treatment and follow-up. Ann Oncol 31(10):1306–1319. 10.1016/j.annonc.2020.07.003 32739407 10.1016/j.annonc.2020.07.003

[CR31] Kasi A, Abbasi S, Handa S, Al-Rajabi R, Saeed A, Baranda J et al (2020) Total neoadjuvant therapy vs standard therapy in locally advanced rectal cancer: a systematic review and meta-analysis. JAMA Netw Open 3(12):e2030097-e. 10.1001/jamanetworkopen.2020.30097 33326026 10.1001/jamanetworkopen.2020.30097 PMC7745099

[CR32] Keller DS, Berho M, Perez RO, Wexner SD, Chand M (2020) The multidisciplinary management of rectal cancer. Nat Rev Gastroenterol Hepatol 17(7):414–429. 10.1038/s41575-020-0275-y 32203400 10.1038/s41575-020-0275-y

[CR33] Kim Y, Kim Y, Kwak Y (2021) Factors associated with post-traumatic growth in male patients with rectal cancer: a cross-sectional study. Eur J Oncol Nurs 54:102028. 10.1016/j.ejon.2021.102028 34507151 10.1016/j.ejon.2021.102028

[CR34] Lawday S, Flamey N, Fowler GE, Leaning M, Dyar N, Daniels IR et al (2021) Quality of life in restorative versus non-restorative resections for rectal cancer: systematic review. BJS Open. 10.1093/bjsopen/zrab101 35040944 10.1093/bjsopen/zrab101 PMC8765336

[CR35] Lawday S, Flamey N, Fowler GE, Leaning M, Dyar N, Daniels IR et al (2022) Quality of life in restorative versus non-restorative resections for rectal cancer: systematic review. BJS Open. 10.1093/bjsopen/zrab101 10.1093/bjsopen/zrab101 PMC8765336 35040944

[CR36] Lim RS, Yang TX, Chua TC (2014) Postoperative bladder and sexual function in patients undergoing surgery for rectal cancer: a systematic review and meta-analysis of laparoscopic versus open resection of rectal cancer. Tech Coloproctol 18(11):993–1002. 10.1007/s10151-014-1189-x 25056719 10.1007/s10151-014-1189-x

[CR37] Liu Z, Thong MSY, Doege D, Koch-Gallenkamp L, Bertram H, Eberle A et al (2021) Prevalence of benefit finding and posttraumatic growth in long-term cancer survivors: results from a multi-regional population-based survey in Germany. Br J Cancer 125(6):877–883. 10.1038/s41416-021-01473-z 34215852 10.1038/s41416-021-01473-z PMC8437934

[CR38] Loprinzi CL, Lacchetti C, Bleeker J, Cavaletti G, Chauhan C, Hertz DL et al (2020) Prevention and management of chemotherapy-induced peripheral neuropathy in survivors of adult cancers: ASCO guideline update. J Clin Oncol 38(28):3325–3348. 10.1200/jco.20.01399 32663120 10.1200/JCO.20.01399

[CR39] Musoro JZ, Sodergren SC, Coens C, Pochesci A, Terada M, King MT et al (2020) Minimally important differences for interpreting the EORTC QLQ-C30 in patients with advanced colorectal cancer treated with chemotherapy. Colorectal Dis 22(12):2278–2287. 10.1111/codi.15295 32767619 10.1111/codi.15295

[CR40] Cohen J (1988) Statistical power analysis for the behavioral sciences, 2nd ed. Lawrence Erlbaum Associates Inc., New Jersey, p 13

[CR41] Nolte S, Liegl G, Petersen MA, Aaronson NK, Costantini A, Fayers PM et al (2019) General population normative data for the EORTC QLQ-C30 health-related quality of life questionnaire based on 15,386 persons across 13 European countries, Canada and the Unites States. Eur J Cancer 107:153–163. 10.1016/j.ejca.2018.11.024 30576971 10.1016/j.ejca.2018.11.024

[CR42] Nolte S, Waldmann A, Liegl G, Petersen MA, Groenvold M, Rose M (2020) Updated EORTC QLQ-C30 general population norm data for Germany. Eur J Cancer 137:161–170. 10.1016/j.ejca.2020.06.002 32777715 10.1016/j.ejca.2020.06.002

[CR43] Ochiai K, Bhutiani N, Ikeda A, Uppal A, White MG, Peacock O et al (2024) Total neoadjuvant therapy for rectal cancer: which regimens to use? Cancers (Basel). 10.3390/cancers16112093 38893212 10.3390/cancers16112093 PMC11171181

[CR44] Osoba D, Rodrigues G, Myles J, Zee B, Pater J (1998) Interpreting the significance of changes in health-related quality-of-life scores. J Clin Oncol 16(1):139–144. 10.1200/jco.1998.16.1.139 9440735 10.1200/JCO.1998.16.1.139

[CR45] Petrelli F, Trevisan F, Cabiddu M, Sgroi G, Bruschieri L, Rausa E et al (2020) Total neoadjuvant therapy in rectal cancer: a systematic review and meta-analysis of treatment outcomes. Ann Surg 271(3):440–448. 10.1097/sla.0000000000003471 31318794 10.1097/SLA.0000000000003471

[CR46] Quezada-Diaz FF, Smith JJ, Jimenez-Rodriguez RM, Wasserman I, Pappou EP, Patil S et al (2020) Patient-reported bowel function in patients with rectal cancer managed by a watch-and-wait strategy after neoadjuvant therapy: a case-control study. Dis Colon Rectum 63(7):897–902. 10.1097/dcr.0000000000001646 32217857 10.1097/DCR.0000000000001646 PMC7274891

[CR47] Rausa E, Kelly ME, Bonavina L, O’Connell PR, Winter DC (2017) A systematic review examining quality of life following pelvic exenteration for locally advanced and recurrent rectal cancer. Colorectal Dis 19(5):430–436. 10.1111/codi.13647 28267255 10.1111/codi.13647

[CR48] Rödel C, Graeven U, Fietkau R, Hohenberger W, Hothorn T, Arnold D et al (2015) Oxaliplatin added to fluorouracil-based preoperative chemoradiotherapy and postoperative chemotherapy of locally advanced rectal cancer (the German CAO/ARO/AIO-04 study): final results of the multicentre, open-label, randomised, phase 3 trial. Lancet Oncol 16(8):979–989. 10.1016/s1470-2045(15)00159-x 26189067 10.1016/S1470-2045(15)00159-X

[CR49] Scherer-Trame S, Jansen L, Koch-Gallenkamp L, Arndt V, Chang-Claude J, Hoffmeister M et al (2022) Quality of life, distress, and posttraumatic growth 5 years after colorectal cancer diagnosis according to history of inpatient rehabilitation. J Cancer Res Clin Oncol 148(11):3015–3028. 10.1007/s00432-021-03865-3 34874489 10.1007/s00432-021-03865-3 PMC9508041

[CR50] Seow W, Murshed I, Bunjo Z, Bedrikovetski S, Stone J, Sammour T (2025) Compliance and toxicity of total neoadjuvant therapy in locally advanced rectal cancer: a systematic review and network meta-analysis. Ann Surg Oncol. 10.1245/s10434-025-17421-7 40325300 10.1245/s10434-025-17421-7 PMC12317888

[CR51] Stiggelbout AM, Kunneman M, Baas-Thijssen MCM, Neijenhuis PA, Loor AK, Jägers S et al (2016) The EORTC QLQ-CR29 quality of life questionnaire for colorectal cancer: validation of the Dutch version. Qual Life Res 25(7):1853–1858. 10.1007/s11136-015-1210-5 26711791 10.1007/s11136-015-1210-5 PMC4893365

[CR52] Sullivan GM, Feinn R (2012) Using effect size—or why the P value is not enough. J Grad Med Educ 4(3):279–282. 10.4300/jgme-d-12-00156.1 23997866 10.4300/JGME-D-12-00156.1 PMC3444174

[CR53] Turri G, Ostuzzi G, Vita G, Barresi V, Scarpa A, Milella M et al (2024) Treatment of locally advanced rectal cancer in the era of total neoadjuvant therapy: a systematic review and network meta-analysis. JAMA Netw Open 7(6):e2414702. 10.1001/jamanetworkopen.2024.14702 38833249 10.1001/jamanetworkopen.2024.14702 PMC11151159

[CR54] van der Hout A, Neijenhuijs KI, Jansen F, van Uden-Kraan CF, Aaronson NK, Groenvold M et al (2019) Measuring health-related quality of life in colorectal cancer patients: systematic review of measurement properties of the EORTC QLQ-CR29. Support Care Cancer 27(7):2395–2412. 10.1007/s00520-019-04764-7 30982095 10.1007/s00520-019-04764-7 PMC6541702

[CR55] von Elm E, Altman DG, Egger M, Pocock SJ, Gøtzsche PC, Vandenbroucke JP (2007) The strengthening the reporting of observational studies in epidemiology (STROBE) statement: guidelines for reporting observational studies. Lancet 370(9596):1453–1457. 10.1016/S0140-6736(07)61602-X 18064739 10.1016/S0140-6736(07)61602-X

[CR56] Vonk-Klaassen SM, de Vocht HM, den Ouden ME, Eddes EH, Schuurmans MJ (2016) Ostomy-related problems and their impact on quality of life of colorectal cancer ostomates: a systematic review. Qual Life Res 25(1):125–133. 10.1007/s11136-015-1050-3 26123983 10.1007/s11136-015-1050-3 PMC4706578

[CR57] Whistance RN, Conroy T, Chie W, Costantini A, Sezer O, Koller M et al (2009) Clinical and psychometric validation of the EORTC QLQ-CR29 questionnaire module to assess health-related quality of life in patients with colorectal cancer. Eur J Cancer 45(17):3017–3026. 10.1016/j.ejca.2009.08.014 19765978 10.1016/j.ejca.2009.08.014

[CR58] Wurschi GW, Knippen S, Ernst T, Schneider C, Helfritzsch H, Mothes H et al (2023) Long-term total neoadjuvant therapy leads to impressive response rates in rectal cancer: results of a german single-center cohort. Curr Oncol 30(6):5366–5378 37366890 10.3390/curroncol30060407 PMC10297568

[CR59] Wurschi G, Mäurer M, Aninditha KP, Becker J-N, Bischoff M, Büttner M et al (2024) TNTox—outcome und Toxizität nach totaler neoadjuvanter Therapie beim Rektumkarzinom. Forum 39(4):304–307. 10.1007/s12312-024-01339-4

[CR60] Wurschi GW, Rühle A, Domschikowski J, Trommer M, Ferdinandus S, Becker JN et al (2024) Patient-relevant costs for organ preservation versus radical resection in locally advanced rectal cancer. Cancers (Basel). 10.3390/cancers16071281 38610958 10.3390/cancers16071281 PMC11011197

