# Supplementary material for: Quality of life following total neoadjuvant therapy for rectal cancer
Source: J Cancer Res Clin Oncol. 2025 Oct 25;151(12):304. doi: 10.1007/s00432-025-06347-y (PMC12553667; doi:10.1007/s00432-025-06347-y)
Supplement: Supplementary file 1 — Supplementary file1 (PDF 1292 kb) [file 432_2025_6347_MOESM1_ESM.pdf]

Quality of Life following Total Neoadjuvant Therapy for Rectal Cancer

Supplementary Document

Georg W. Wurschi, Markus Diefenhardt, Justus Kaufmann, Hai Minh Ha, Melanie Schneider, Daphne Schepers von Ohlen, Maren Schöneich, Adrianna Cieslak, Alina Depardon, Jan-Niklas Becker, Alexander Rühle, Felix Ehret, Maximilian Römer, Florian Rißner, Andreas Hinz, and Klaus Pietschmann

Table of contents

S1. Table: Participating centers 2
S2. Table: Treatment failures 3
S3. Table: Chronic toxicity after TNT at the last follow-up 4
S4. Table: Bowel/Defecation related symptoms (QLQ-CR29) 7
S5. Table: Internal consistency of Defecation/Stoma-related problems (CR-29) 8
S6. Table: Distribution of patients within age groups 9
S7. Table: QLQ-C30 and CR29 mean values of the TNTox and reference cohorts 12
S8. Table: Known group comparisons for quality of life scores 14
S9. Table: Association of quality of life scores with continuous factors 17
References 18

S1. Table: Participating centers

Detailed overview of all centers participating in this study and the number of included patients per center.

Center	Number of Patients
Jena University Hospital (Jena / Germany)	28
University Hospital / Goethe University Frankfurt (Frankfurt / Germany)	19
University Medical Center of the Johannes-Gutenberg-University Mainz (Mainz / Germany)	6
Otto von Guericke Universität Magdeburg (Magdeburg / Germany)	5
University Hospital Carl Gustav Carus Dresden (Dresden / Germany)	4
University Medical Center Schleswig-Holstein / Campus Lübeck (Lübeck / Germany)	4
University Medical Center Schleswig-Holstein / Campus Kiel (Kiel / Germany)	2
University Medicine Mannheim, Medical Faculty Mannheim (Mannheim / Germany)	2
Hannover Medical School (Hannover / Germany)	1
Universitätsklinikum Erlangen, Friedrich-Alexander-Universität Erlangen-Nürnberg (Erlangen / Germany)	1
**Total**	72

S2. Table: Treatment failures

Localization of treatment failures (A) and ongoing treatments at follow-up (B). The number of patients (n) and the relative frequency (%) receiving a treatment are provided; a combination of different therapies per patient was possible.

**(A)**	**N**		**n (%)**		**n (%)**
**Relapse**	71	Yes	12 (16.9%)	No	59 (83.1%)
**Localization***		Local recurrence (in-field)	6 (8.5%)		
		Localized, out-of-field:	2 (2.8%)		
		Distant failure (e.g., liver/lung metastasis):	8 (11.26%)		

**(B) Treatment for relapse (at follow-up)***	**N**		**n (%)**		**n (%)**
	71	None	2 (2.8%)		
**Systemic treatment**		Completed (> 3 months)	2 (2.8%)	Ongoing / recently completed (<3 months):	2 (2.8%)
**Resection**		Completed (> 3 months)	2 (2.8%)	Ongoing / recently completed (<3 months):	5 (7.0%)
**Local (re)-irradiation**		Completed (> 3 months)	1 (1.4%)	Ongoing / recently completed (<3 months):	1 (1.4%)
**Chemo-(re)-irradiation**		Completed (> 3 months)	1 (1.4%)	Ongoing / recently completed (<3 months):	0 (0.0%)

Footnotes:
* Since multiple responses were possible, the total number of relapse localizations and ongoing treatments exceeds the number of patients with observed relapses.

S3. Table: Chronic toxicity after TNT at the last follow-up

Toxicity is classified according to CTCAE v5.0. Absolute (n) and relative frequencies (%) are provided. Furthermore, the number of patients (N) per group with available data for the respective characteristic is given. Due to rounding, percentages may not add up to exactly 100%.

**Frequency Tables**			
**Fatigue**	n	%	Cumulative %
**none / Grade 0**	37	54.4	54.4
**slight / CTC Grade I**	22	32.4	86.8
**moderate / CTC Grade II**	8	11.8	98.5
**severe / CTC Grade III**	1	1.5	100.0
**n**	68		
**Pain**			
**none / Grade 0**	46	67.6	67.6
**slight / CTC Grade I**	14	20.6	88.2
**moderate / CTC Grade II**	7	10.3	98.5
**severe / CTC Grade III**	1	1.5	100.0
**n**	68		
**Nausea / Vomiting**			
**none / Grade 0**	63	91.3	91.3
**slight / CTC Grade I**	4	5.8	97.1
**moderate / CTC Grade II**	2	2.9	100.0
**n**	69		
**Diarrhea**			
**none / Grade 0**	52	75.4	75.4
**mild / CTC Grade I**	10	14.5	89.9
**moderate / CTC Grade II**	5	7.2	97.1
**severe / CTC Grade III**	2	2.9	100.0
**n**	69		
**Proctitis**			
**none / Grade 0**	58	84.1	84.1
**mild / CTC Grade I**	7	10.1	94.2
**moderate / CTC Grade II**	1	1.4	95.7
**severe / CTC Grade III**	3	4.3	100.0
**n**	69		
**Cystitis**			
**none / Grade 0**	62	91.2	91.2
**mild / CTC Grade I**	4	5.9	97.1
**moderate / CTC Grade II**	2	2.9	100.0
**n**	68		
**Fecal incontinence**			
**none / Grade 0**	56	83.6	83.6
**mild / CTC Grade I**	6	9.0	92.5
**moderate / CTC Grade II**	3	4.5	97.0

**severe / CTC Grade III**	2	3.0	100.0
**n**	67		
**Constipation / Ileus**			
**none / Grade 0**	63	91.3	91.3
**mild / CTC Grade I**	5	7.2	98.6
**moderate / CTC Grade II**	1	1.4	100.0
**n**	69		
**Bowel stenosis**			
**none / Grade 0**	67	98.5	98.5
**severe / CTC Grade III**	1	1.5	100.0
**n**	68		
**Urine incontinence**			
**none / Grade 0**	67	98.5	98.5
**mild / CTC Grade I**	1	1.5	1100.0
**n**	68		
**Dermatitis**			
**none / Grade 0**	64	92.8	92.8
**slight / CTC Grade I**	5	7.2	100.0
**n**	69		
**Mucositis**			
**none / Grade 0**	68	98.6	98.6
**mild / CTC Grade I**	1	1.4	100.0
**n**	69		
**Fistula (any localization)**			
**none / Grade 0**	66	95.7	95.7
**mild / CTC Grade I**	1	1.4	97.1
**moderate / CTC Grade II**	1	1.4	98.6
**severe / CTC Grade III**	1	1.4	100.0
**n**	69		
**Bleeding**			
**none / Grade 0**	64	94.1	94.1
**mild / CTC Grade I**	4	5.9	100.0
**n**	68		
**Polyneuropathy**			
**none / Grade 0**	28	40.6	40.6
**mild / CTC Grade I**	22	31.9	72.5
**moderate / CTC Grade II**	14	20.3	92.8
**severe / CTC Grade III**	5	7.2	100.0
**n**	69		
**Dyspnea**			
**none / Grade 0**	64	92.8	92.8
**mild / CTC Grade I**	3	4.3	97.1
**moderate / CTC Grade II**	2	2.9	100.0
**n**	69		

**Infections**			
**none / Grade 0**	69	100.0	100.0
**n**	69		
**Liver damage / Hepatitis**			
**none / Grade 0**	68	100.0	100.0
**n**	68		
**Kidney damage**			
**none / Grade 0**	67	100.0	100.0
**n**	67		
**Erectile dysfunction**			
**none / Grade 0**	29	64.4	64.4
**mild / CTC Grade I**	2	4.4	68.9
**moderate / CTC Grade II**	5	11.1	80
**severe / CTC Grade III**	9	20.0	100.0
**n**	45		
**Dyspareunia**			
**none / Grade 0**	14	93.3	93.3
**severe / CTC Grade III**	1	6.7	100.0
**n**	15		

S4. Table: Bowel/Defecation related symptoms (QLQ-CR29)

Descriptive statistics of bowel/defecation-related symptoms of the QLQ-CR29 questionnaire, stratified per presence of a stoma. All scales represent symptom scales, i.e., a higher value indicates more symptoms. The number of returned questionnaires (n) is provided along with Mean, standard deviation (SD), and Median with 1st and 3rd quartile (Q1, Q3). 95% confidence intervals were obtained from 1000 bootstrap samples.

**Patients with stoma**								
		Mean	95% Confidence Interval Mean		SD	Median	Q1	Q3
	n		Lower	Upper				
**Flatulence**	24	20.8	12.5	30.6	21.6	33.3	0.0	33.3
**Fecal incontinence**	24	12.5	5.6	20.8	19.2	0.0	0.0	33.3
**Sore skin**	24	26.4	15.3	37.5	27.8	33.3	0.0	41.7
**Stool frequency**	23	22.5	13.0	32.6	24.4	16.7	0.0	33.3
**Embarrassment**	25	25.3	12.0	40.0	36.4	0.0	0.0	33.3

**Patients without stoma**								
		Mean	95% Confidence Interval Mean		SD	Median	Q1	Q3
	n		Lower	Upper				
**Flatulence**	41	41.5	31.7	50.4	31.4	33.3	33.3	66.7
**Fecal incontinence**	39	33.3	24.8	43.6	30.6	33.3	0.0	33.3
**Sore skin**	39	26.5	17.1	37.6	33.5	0.0	0.0	33.3
**Stool frequency**	39	36.8	28.2	44.9	27.6	33.3	16.7	50.0
**Embarrassment**	38	36.0	22.8	47.4	39.0	33.3	0.0	66.7

S5. Table: Internal consistency of Defecation/Stoma-related problems (CR-29)

Assessment of internal consistency of the multi-item scale “bowel/defecation related symptoms” (Items 49-54) of the QLQ-CR29 questionnaire, which was proposed by Stiggelbout et al [1]. The multi-item scale presents a symptom scale, i.e., a higher value indicates more symptoms. Internal consistency (A) was assessed with Cronbach’s α and McDonald’s ω. Both parameters were evaluated in the context of the influence of dropping single items from the scale (B). Spearman’s ρ was furthermore calculated between this multi-item scale and other established multi-item scales of the QLQ-C30 (i.e., the ‘global health scale’ and the ‘summary score’ [2]) and the QLQ-CR29 (i.e., ‘body image’ and ‘blood and mucus in stool (BMS)’ as well as selected single items related with bowel function (i.e., QLQ-C30 ‘diarrhea’) (C). 95% confidence intervals were obtained from 1000 bootstrap samples.

**(A) Scale Reliability Statistics**				
	Estimate	Std. Error	95% CI	
**Coefficient**			Lower	Upper
**Coefficient ω**	0.847	0.035	0.771	0.901
**Coefficient α**	0.845	0.037	0.758	0.899

**(B) Individual Item Reliability Statistics**						
	Coefficient ω (if item dropped)			Coefficient α (if item dropped)		
**Item**	Estimate	Lower 95% CI	Upper 95% CI	Estimate	Lower 95% CI	Upper 95% CI
**Stool frequency (Item 52)**	0.810	0.715	0.875	0.807	0.695	0.871
**Stool frequency (Item 53)**	0.818	0.723	0.883	0.815	0.707	0.879
**Embarassment (Item 54)**	0.814	0.721	0.880	0.810	0.702	0.877
**Fecal incontinence (Item 50)**	0.816	0.723	0.880	0.810	0.705	0.875
**Flatulence (Item 49)**	0.839	0.764	0.897	0.837	0.758	0.895
**Sore skin (Item 51)**	0.839	0.734	0.901	0.837	0.725	0.900

**(C) Spearman's Correlations**			**Spearman's ρ**			**p**
				Lower 95% CI	Upper 95% CI	
**Defecation/Stoma Problems (QLQ-CR29)⁻**	-	Global Health Scale (QLQ-C30)⁺	-0.452	-0.628	-0.234	< .001*
**Defecation/Stoma Problems (QLQ-CR29)⁻**	-	Sum Score (QLQ-C30)⁺	-0.585	-0.750	-0.387	< .001*
**Defecation/Stoma Problems (QLQ-CR29)⁻**	-	Body Image (QLQ-CR29)⁺	-0.472	-0.637	-0.276	< .001*
**Defecation/Stoma Problems (QLQ-CR29)⁻**	-	Blood/mucus in stool (QLQ-CR29)⁻	0.241	0.008	0.454	0.068
**Defecation/Stoma Problems (QLQ-CR29)⁻**	-	Diarrhea (QLQ-C30)⁻	0.478	0.244	0.663	< .001*

Note. Confidence intervals based on 1000 bootstrap replicates.

Footnotes:
+ Function scale, i.e., higher values indicate better function.
- Symptom scale, i.e., higher values indicate more symptoms.
* Significant correlations at unadjusted (p<0.05) and Bonferroni-adjusted (p<0.01) thresholds.

S6. Table: Distribution of patients within age groups

Distribution of patients stratified per age group (in 10-year intervals) and sex within the TNTox cohort. These relative frequencies were applied for standardization of the reference values for the German general population, i.e., the relative frequency of patients per subgroup (A) was used to calculate the weighted health-related quality of life (HRQoL) scores based on the respective HRQoL per subgroup as provided by Hinz et al. [3] (B). Abbreviations: mean (M), standard deviation (SD),

**(A) Age-group (10 years interval) × sex**			
	Frequency (n)	Percent	Cumulative Percent
Male, 30-39	1	1.4	1.4
Male, 40-49	4	5.6	6.9
Male, 50-59	13	18.1	25.0
Male, 60-69	25	34.7	59.7
Male, 70+	11	15.3	75.0
Female, 40-49	1	1.4	76.4
Female, 50-59	3	4.2	80.6
Female, 60-69	9	12.5	93.1
Female, 70+	5	6.9	100.0
Total N	72	100.0	

Table B (continued) – see next page

(B) Quality of life per age group, derived from Hinz et al. [3]

	Men							Women							Total	**Standardized to TNTox**
	All	18-29	30-39	40-49	50-59	60-69	≥ 70	All	18-29	30-39	40-49	50-59	60-69	≥ 70		
**N**	1139	191	158	177	227	201	185	1.309	208	185	219	272	215	210	2448	
**Functioning scales**																
**Physical M**	92.9	97.8	98.5	96.3	93.6	92.3	79.7	91.5	97.0	97.3	95.0	92.9	89.2	78.0	92.2	**88.2**
**(SD)**	14.6	7.5	6.5	11.5	13.9	12.3	21.4	15.5	10.1	9.7	12.5	13.7	14.6	20.6	15.1	**14.7**
**Role M**	90.9	95.6	97.1	93.2	91.1	90.7	78.3	89.9	97.3	94.0	92.6	91.9	87.5	76.2	90.4	**86.4**
**(SD)**	19.8	13.8	12.8	20.1	19.4	16.6	26.8	20.6	11.4	17.8	17.0	18.5	21.1	27.5	20.2	**20**
**Emotional M**	83.9	84.4	89.0	83.7	80.9	85.6	81.0	83.2	86.0	85.6	81.9	82.2	83.5	80.2	83.5	**81.9**
**(SD)**	20.1	21.6	16.5	21.2	21.5	18.4	19.0	19.3	19.8	17.7	18.8	20.6	16.9	20.8	19.7	**19**
**Cognitive M**	93.7	96.3	98.2	94.6	94.3	94.2	85.1	93.4	96.9	96.0	93.5	94.5	93.0	86.3	93.5	**90.8**
**(SD)**	14.5	11.6	6.9	13.0	14.0	12.8	20.9	14.6	10.3	13.0	15.3	13.4	13.1	18.7	14.5	**14.6**
**Social M**	93.6	96.3	97.5	94.0	93.5	94.1	86.8	93.3	97.1	94.5	94.1	93.6	93.2	87.2	93.4	**90.9**
**(SD)**	17.2	13.2	10.2	18.9	17.7	16.2	22.1	17.1	11.6	16.1	15.2	17.0	16.7	22.6	17.2	**17.8**
**Global health / QoL M**	75.5	82.9	83.3	80.0	74.7	71.6	62.3	74.5	84.3	82.6	77.7	74.4	69.0	60.2	75.0	**69.3**
**(SD)**	19.8	17.9	15.7	17.5	19.0	18.5	21.5	19.4	17.2	15.4	17.6	17.9	17.5	20.0	19.6	**18.7**
**Symptoms**																
**Fatigue M**	14.5	10.8	6.9	10.6	14.5	14.5	28.7	16.4	10.1	12.1	14.4	15.6	18.3	27.9	15.5	**17.7**
**(SD)**	21.8	20.7	12.5	18.8	22.2	19.3	27.0	21.4	17.4	20.0	19.4	21.1	19.3	25.9	21.6	**21.2**
**Nausea / Vomiting M**	1.9	2.1	0.4	0.7	2.0	2.4	3.3	2.4	2.0	2.7	1.8	1.6	2.6	3.9	2.2	**2.4**
**(SD)**	7.9	10.0	2.6	5.0	8.3	8.7	9.2	9.6	8.3	11.3	7.8	7.9	10.0	12.0	8.9	**8.7**
**Pain M**	16.4	7.8	6.3	13.1	16.8	20.6	32.3	17.0	7.2	9.0	13.2	16.0	24.2	31.6	16.7	**21.9**
**(SD)**	24.3	18.7	16.8	24.9	24.1	23.3	26.3	24.2	17.6	20.6	20.6	24.7	24.9	26.0	24.2	**24.1**
**Dyspnea M**	7.8	2.3	1.3	3.2	8.1	8.5	22.3	7.2	2.1	3.4	5.0	6.4	9.3	16.7	7.5	**10.7**
**(SD)**	20.0	10.8	8.3	12.6	21.2	19.2	29.6	18.7	9.3	13.2	16.0	17.9	20.0	26.7	19.3	**21**
**Insomnia M**	11.6	6.6	6.5	9.8	13.2	11.9	20.4	13.0	5.6	7.7	10.6	15.8	15.7	21.3	12.4	**14.4**
**(SD)**	22.9	20.6	17.8	22.0	24.1	21.9	26.7	23.6	16.2	18.9	21.8	26.4	24.3	27.1	23.3	**23.6**
**Appetite loss M**	3.2	2.1	1.0	2.4	4.3	2.0	7.1	4.2	3.4	3.4	4.4	3.2	4.0	7.0	3.8	**3.9**
**(SD)**	12.5	11.1	8.8	12.3	15.3	7.9	15.7	13.9	14.4	14.5	13.4	12.1	12.3	16.8	13.3	**12**

**Constipation M**	1.3	0.7	0.6	0.9	1.6	0.7	2.9	3.1	1.4	1.6	2.9	2.9	4.0	5.6	2.2	**2.1**
**(SD)**	7.6	4.8	5.9	6.6	8.4	4.7	12.2	12.1	7.6	9.4	10.9	12.5	13.9	15.8	10.3	**8.9**
**Diarrhea M**	2.1	1.0	0.6	1.7	2.6	2.8	3.2	2.9	3.5	3.4	4.0	1.5	2.3	3.0	2.5	**2.6**
**(SD)**	10.1	8.3	4.6	8.9	11.4	12.8	11.1	12.7	13.4	15.4	15.8	8.5	10.7	12.1	11.6	**11.4**
**Financial Difficulties M**	4.8	2.6	1.9	5.1	5.5	3.5	10.1	4.8	2.6	2.0	5.6	4.5	4.8	9.3	4.8	**5.5**
**(SD)**	16.9	13.6	10.2	20.0	19.0	13.5	20.4	16.3	11.6	11.1	17.6	17.9	14.2	21.0	16.6	**16.6**

S7. Table: QLQ-C30 and CR29 mean values of the TNTox and reference cohorts

Comparison of quality of life within the TNTox cohort and reference values from the German general population [3] as well as from colorectal cancer (CRC) patients, who underwent curative-intent treatment within 12 months from diagnosis [4]. For the German reference values, a standardization according to the distribution of age and sex within the TNTox cohort was performed (see also Supplement S6). Absolute frequencies (n), mean together with standard deviation (SD), and the distribution of the mean based on 1000 bootstrap samples in the TNTox cohort are provided. Cohen's d is provided for the determination of the effect size between the TNTox cohort and the two reference groups; the standardized Mean / SD values of the German general population were used. Note that the TNTox cohort is evaluated against the reference cohorts, resulting in negative effect sizes for lower mean values in the TNTox cohort and vice versa. Higher values indicate better function in functioning scales, whereas lower values indicate lower symptoms in the symptom scales.

	**TNTox cohort**					**German general population**							**EORTC CRC patients (curative intent treatment)**				
**Scale**	Mean	95%-CI of Mean		SD	n	Mean	SD	Standard-ized Mean	Standard-ized SD	n	Difference in means	Cohen's d	Mean	SD	n	Difference in means	Cohen's d
**QLQ-C30**		Lower	Upper														
**Function scales**																	
**Global health**	64.1	58.6	69.2	23.1	69	75.0	19.6	69.3	14.7	2448	-5.2	-0.27	64.0	23.0	275	0.1	0.00
**Physical**	76.7	70.9	81.6	23.6	72	92.2	15.1	88.2	20.0	2448	-11.5	-0.53	77.0	23.0	275	-0.3	-0.01
**Role**	61.7	54.3	68.6	31.6	70	90.4	20.2	86.4	19.0	2448	-24.7	-0.95	68.0	34.0	275	-6.3	-0.19
**Emotional**	75.4	68.3	81.3	26.7	70	83.5	19.7	81.9	14.6	2448	-6.5	-0.30	74.0	25.0	275	1.4	0.05
**Cognitive**	82.1	76.3	87.2	23.3	69	93.5	14.5	90.8	17.8	2448	-8.7	-0.42	81.0	21.0	275	1.1	0.05
**Social**	68.1	60.8	75.0	31.2	68	93.4	17.2	90.9	18.7	2448	-22.8	-0.89	70.0	30.0	275	-1.9	-0.06
**Symptom Scales**																	
**Fatigue**	29.0	23.2	35.1	26.4	71	15.5	21.6	17.7	21.2	2448	11.3	0.47	37.0	27.0	275	-8.0	-0.30
**Nausea and vomiting**	5.0	2.6	8.3	12.2	70	2.2	8.9	2.4	8.7	2448	2.6	0.24	9.0	18.0	275	-4.0	-0.26
**Pain**	19.8	14.3	26.0	25.9	70	16.7	24.2	21.9	24.1	2448	-2.1	-0.08	24.0	28.0	275	-4.2	-0.16
**Dyspnea**	16.9	12.2	22.5	23.8	71	7.5	19.3	10.7	21.0	2448	6.2	0.28					
**Insomnia**	28.6	21.1	36.6	33.5	71	12.4	23.3	14.4	23.6	2448	14.2	0.49					
**Appetite Loss**	13.1	6.6	19.3	27.9	71	3.8	13.3	3.9	12.0	2448	9.2	0.43					
**Constipation**	13.1	7.5	19.2	24.9	71	2.2	10.3	2.1	8.9	2448	11.0	0.59					
**Diarrhea**	21.3	14.0	29.0	30.8	69	2.5	11.6	2.6	11.4	2448	18.7	0.80					
**Financial difficulties**	17.6	11.3	24.5	29.1	68	4.8	16.6	5.5	16.6	2448	12.1	0.51					

(Table S7 – continued)

**QLQ-CR29**										
**Function scales**										
**Body image**	77.0	71.3	82.9	25.6	69	81.0	26.0	275	-4.0	-0.16
**Anxiety**	60.4	52.7	68.6	33.0	69	59.0	33.0	275	1.4	0.04
**Weight**	78.3	72.0	84.1	25.5	69	80.0	28.0	275	-1.7	-0.06
**Sexual interest (men)**	42.4	34.0	50.7	32.1	48	23.0	30.0	275	19.4	0.62
**Sexual interest (women)**	22.2	11.1	35.6	24.1	15	12.0	23.0	275	10.2	0.43
**Symptom Scales**										
**Stool frequency**	31.5	25.0	38.7	27.2	62	22.0	25.0	275	9.5	0.36
**Urinary frequency**	37.9	31.9	43.7	25.9	69	37.0	27.0	275	0.9	0.03
**Blood and mucus in stool**	7.9	5.1	11.0	12.9	65	7.0	15.0	275	0.9	0.06
**Urinary incontinence**	11.9	7.5	15.9	19.0	67	9.0	21.0	275	2.9	0.14
**Dysuria**	7.5	3.8	12.2	18.9	71	5.0	15.0	275	2.5	0.15
**Abdominal pain**	11.7	7.0	16.4	20.4	71	18.0	26.0	275	-6.3	-0.27
**Buttock pain**	23.0	17.4	28.6	26.8	71	14.0	26.0	275	9.0	0.34
**Bloating**	21.4	15.2	28.1	26.6	70	20.0	27.0	275	1.4	0.05
**Dry mouth**	16.9	11.3	22.5	24.5	71	26.0	29.0	275	-9.1	-0.34
**Hair loss**	16.0	10.3	22.1	25.7	71	7.0	19.0	275	9.0	0.40
**Taste**	15.5	8.9	23.0	29.2	71	14.0	27.0	275	1.5	0.05
**Flatulence**	33.8	27.2	41.0	29.8	65	27.0	29.0	275	6.8	0.23
**Fecal incontinence**	25.4	18.5	32.8	28.5	63	12.0	23.0	275	13.4	0.52
**Sore skin**	26.5	19.0	34.4	31.2	63	17.0	27.0	275	9.5	0.33
**Embarrassment**	31.7	22.8	41.8	38.1	63	19.0	30.0	275	12.7	0.37
**Stoma care problems**	12.0	4.0	21.3	23.3	25	16.0	22.0	275	-4.0	-0.18
**Impotence**	63.0	51.4	73.9	38.6	46	34.0	41.0	275	29.0	0.73
**Dyspareunia**	19.4	5.6	36.1	30.0	12	9.0	21.0	275	10.4	0.40

Footnotes:
* In contrast to the original publication, values were converted to a function scale format according to the questionnaire’s handbook for comparison.

S8. Table: Known group comparisons for quality of life scores

Comparison of multi-item scales for predefined subgroups with the Mann-Whitney U test (A) and the respective group characteristics (B). Abbreviations: Non-operative management, NOM; standard error, SE.

**(A) Mann-Whitney U test**				
**Non-operative management (NOM)**				
	U	p	Rank-Biserial Correlation	SE Rank-Biserial Correlation
**Global Health Scale (QLQ-C30)⁺**	444.0	0.678	-0.065	0.155
**Summary Score (QLQ-C30)⁺**	446.5	0.633	0.079	0.160
**Body Image (QLQ-CR29)⁺**	439.0	0.623	-0.076	0.155
**Blood/mucus in stool (QLQ-CR29)⁻**	360.5	0.396	-0.116	0.163
**Defecation/Stoma Problems (QLQ-CR29)⁻**	291.5	0.345	-0.167	0.174

**Stoma**				
	U	p	Rank-Biserial Correlation	SE Rank-Biserial Correlation
**Global Health Scale (QLQ-C30)⁺**	666.5	0.073	0.262	0.147
**Summary Score (QLQ-C30)⁺**	514.0	0.720	0.054	0.148
**Body Image (QLQ-CR29)⁺**	655.5	0.135	0.214	0.146
**Blood/mucus in stool (QLQ-CR29)⁻**	542.5	0.300	0.130	0.149
**Defecation/Stoma Problems (QLQ-CR29)⁻**	641.5	0.034*	0.316	0.148

**Sex**				
	U	p	Rank-Biserial Correlation	SE Rank-Biserial Correlation
**Global Health Scale (QLQ-C30)⁺**	356.5	0.158	-0.223	0.158
**Summary Score (QLQ-C30)⁺**	338.5	0.358	-0.153	0.163
**Body Image (QLQ-CR29)⁺**	365.0	0.187	-0.205	0.158
**Blood/mucus in stool (QLQ-CR29)⁻**	445.5	0.504	0.092	0.163
**Defecation/Stoma Problems (QLQ-CR29)⁻**	449.0	0.606	0.085	0.160

**Chronic toxicity grade ≥2**				
	U	p	Rank-Biserial Correlation	SE Rank-Biserial Correlation
**Global Health Scale (QLQ-C30)⁺**	788.5	0.002**	0.449	0.142
**Summary Score (QLQ-C30)⁺**	724.0	<0.001**	0.508	0.147
**Body Image (QLQ-CR29)⁺**	680.5	0.073	0.250	0.142
**Blood/mucus in stool (QLQ-CR29)⁻**	386.0	0.116	-0.196	0.147
**Defecation/Stoma Problems (QLQ-CR29)⁻**	257.5	0.003**	-0.445	0.148

Table (A) – continued				
**Relapse**				
	U	p	Rank-Biserial Correlation	SE Rank-Biserial Correlation
**Global Health Scale (QLQ-C30)+**	321.5	0.749	-0.060	0.183
**Summary Score (QLQ-C30)+**	250.5	0.471	-0.141	0.190
**Body Image (QLQ-CR29)+**	271.0	0.249	-0.208	0.183
**Blood/mucus in stool (QLQ-CR29)-**	299.5	0.597	0.089	0.197
**Defecation/Stoma Problems (QLQ-CR29)-**	365.0	0.193	0.252	0.190

**(B) Descriptive parameters of group comparisons**					
**NOM**		n	Mean	SD	Mean Rank
**Global Health Scale (QLQ-C30)+**	No	46	78.5	19.0	33.2
	Yes	18	76.0	19.3	30.7
**Summary Score (QLQ-C30)+**	No	50	63.2	24.1	34.4
	Yes	19	66.7	20.8	36.6
**Body Image (QLQ-CR29)+**	No	50	75.3	27.8	34.3
	Yes	19	81.3	18.5	36.9
**Blood/mucus in stool (QLQ-CR29)-**	No	48	7.3	12.8	32.0
	Yes	17	9.8	13.3	35.8
**Defecation/Stoma Problems (QLQ-CR29)-**	No	50	28.5	23.2	31.3
	Yes	14	34.1	22.9	36.7
**Stoma**		n	Mean	SD	Mean Rank
**Global Health Scale (QLQ-C30)+**	No	39	78.3	19.0	33.2
	Yes	25	77.1	19.3	31.4
**Summary Score (QLQ-C30)+**	No	44	67.8	21.3	37.9
	Yes	24	56.6	25.1	28.9
**Body Image (QLQ-CR29)+**	No	45	80.7	22.9	37.6
	Yes	24	69.9	29.2	30.2
**Blood/mucus in stool (QLQ-CR29)-**	No	40	9.2	13.0	34.8
	Yes	24	6.3	12.8	30.5
**Defecation/Stoma Problems (QLQ-CR29)-**	No	39	35.0	24.7	36.4
	Yes	25	21.5	18.0	26.3
**Sex**		n	Mean	SD	Mean Rank
**Global Health Scale (QLQ-C30)+**	female (f)	17	73.6	19.7	28.9
	male (m)	47	79.4	18.6	33.8
**Summary Score (QLQ-C30)+**	female (f)	18	57.4	25.5	29.3
	male (m)	51	66.5	22.0	37.0
**Body Image (QLQ-CR29)+**	female (f)	18	72.2	22.6	29.8
	male (m)	51	78.6	26.6	36.8
**Blood/mucus in stool (QLQ-CR29)-**	female (f)	17	8.8	12.0	35.2
	male (m)	48	7.6	13.3	32.2

**Defecation/Stoma Problems (QLQ-CR29)-**	female (f)	18	31.5	22.7	34.4
	male (m)	46	29.0	23.5	31.7

Table (B) - continued

**Chronic toxicity grade ≥2**					
	Group	n	Mean	SD	Mean Rank
**Global Health Scale (QLQ-C30)+**	No	30	84.5	18.5	40.7
	Yes	32	71.5	17.4	24.6
**Summary Score (QLQ-C30)+**	No	34	73.3	20.8	42.7
	Yes	32	55.2	21.2	27.2
**Body Image (QLQ-CR29)+**	No	33	81.8	23.9	38.9
	Yes	33	70.7	27.0	30.2
**Blood/mucus in stool (QLQ-CR29)-**	No	32	5.7	10.9	30.4
	Yes	30	11.1	14.7	36.8
**Defecation/Stoma Problems (QLQ-CR29)-**	No	29	20.8	18.8	25.2
	Yes	32	38.7	24.3	39.6

**Relapse**					
	Group	n	Mean	SD	Mean Rank
**Global Health Scale (QLQ-C30)+**	No	53	77.7	17.6	31.7
	Yes	11	78.4	25.5	36.2
**Summary Score (QLQ-C30)+**	No	57	64.0	21.9	34.6
	Yes	12	64.6	29.3	36.7
**Body Image (QLQ-CR29)+**	No	57	75.6	25.5	33.8
	Yes	12	83.3	26.2	40.9
**Blood/mucus in stool (QLQ-CR29)-**	No	55	7.9	11.9	33.4
	Yes	10	8.3	18.0	30.6
**Defecation/Stoma Problems (QLQ-CR29)-**	No	53	31.1	22.6	33.9
	Yes	11	23.3	25.6	25.8

Footnotes:
+ Function scale, i.e., higher values indicate better function.
- Symptom scale, i.e., higher values indicate more symptoms.
* Significant correlations at unadjusted (p<0.05) threshold.
** Significant correlations at unadjusted (p<0.05) and Bonferroni-adjusted (p<0.01) thresholds.

S9. Table: Association of quality of life scores with continuous factors

Analysis of the correlation between multi-item scales with predefined continuous factors using Spearman's correlations. 95% confidence intervals (CI) are based on 1000 bootstrap samples. The respective sample size per subscale (n) is given.

**Spearman's Correlations**						
**Variable**		Global Health Scale (QLQ-C30)+	Summary Score (QLQ-C30)+	Body Image (QLQ-CR29)+	Blood/mucus in stool (QLQ-CR29)-	Defecation/Stoma Problems (QLQ-CR29)-
**Age (years)**	n	69	64	69	65	64
	Spearman's ρ	0.128	0.193	0.253	0.084	-0.234
	p-value	0.293	0.127	0.036*	0.507	0.063
	Lower 95% CI	-0.124	-0.032	0.021	-0.174	-0.466
	Upper 95% CI	0.374	0.419	0.455	0.328	0.01
**Duration of TNT (months)**	n	69	64	69	65	64
	Spearman's ρ	-0.251	-0.276	0.119	0.163	0.020
	p-value	0.037*	0.027*	0.331	0.194	0.874
	Lower 95% CI	-0.456	-0.504	-0.121	-0.068	-0.218
	Upper 95% CI	-0.042	-0.042	0.369	0.38	0.26
**Interval to follow-up (months)**	n	69	64	69	65	64
	Spearman's ρ	0.091	0.122	-0.056	-0.077	0.106
	p-value	0.457	0.338	0.647	0.544	0.403
	Lower 95% CI	-0.171	-0.143	-0.296	-0.303	-0.145
	Upper 95% CI	0.332	0.365	0.188	0.178	0.348
**KPS at follow-up (%)**	n	62	59	63	58	58
	Spearman's ρ	0.495	0.436	0.227	-0.038	-0.115
	p-value	< .001**	< .001**	0.074	0.776	0.390
	Lower 95% CI	0.249	0.176	-0.026	-0.279	-0.383
	Upper 95% CI	0.694	0.652	0.467	0.218	0.152

Footnotes:
+ Function scale, i.e., higher values indicate better function.
- Symptom scale, i.e., higher values indicate more symptoms.
* Significant correlations at unadjusted (p<0.05) threshold.
** Significant correlations at unadjusted (p<0.05) and Bonferroni-adjusted (p<0.01) thresholds.

References

1. Stiggelbout, A.M., et al., *The EORTC QLQ-CR29 quality of life questionnaire for colorectal cancer: validation of the Dutch version.* Quality of Life Research, 2016. **25**(7): p. 1853-1858.
2. Giesinger, J.M., et al., *Replication and validation of higher order models demonstrated that a summary score for the EORTC QLQ-C30 is robust.* Journal of Clinical Epidemiology, 2016. **69**: p. 79-88.
3. Hinz, A., S. Singer, and E. Brähler, *European reference values for the quality of life questionnaire EORTC QLQ-C30: Results of a German investigation and a summarizing analysis of six European general population normative studies.* Acta Oncologica, 2014. **53**(7): p. 958-965.
4. Whistance, R.N., et al., *Clinical and psychometric validation of the EORTC QLQ-CR29 questionnaire module to assess health-related quality of life in patients with colorectal cancer.* European Journal of Cancer, 2009. **45**(17): p. 3017-3026.
